# Analysis of genetic diversity of *Xanthomonas oryzae* pv. *oryzae* populations in Taiwan

**DOI:** 10.1038/s41598-018-36575-x

**Published:** 2019-01-22

**Authors:** Chih-Cheng Chien, Mei-Yi Chou, Chun-Yi Chen, Ming-Che Shih

**Affiliations:** 1 Molecular and Biological Agricultural Sciences Program, Taiwan International Graduate Program, Academia Sinica, Taipei, 115 Taiwan; 2 Agricultural Biotechnology Research Center, Academia Sinica, Taipei, 115 Taiwan; 3 Graduate Institute of Biotechnology, National Chung Hsing University, Taichung, 402 Taiwan; 4 Bioinformatics Program, Taiwan International Graduate Program, Academia Sinica, Taipei, 115 Taiwan; 5 Institute of Biomedical Informatics, National Yang-Ming University, Taipei, Taiwan

## Abstract

Rice bacterial blight caused by *Xanthomonas oryzae* pv. *oryzae* (*Xoo*) is a major rice disease. In Taiwan, the tropical indica type of *Oryza sativa* originally grown in this area is mix-cultivated with the temperate japonica type of *O*. *sativa*, and this might have led to adaptive changes of both rice host and *Xoo* isolates. In order to better understand how *Xoo* adapts to this unique environment, we collected and analyzed fifty-one *Xoo* isolates in Taiwan. Three different genetic marker systems consistently identified five groups. Among these groups, two of them had unique sequences in the last acquired ten spacers in the clustered regularly interspaced short palindromic repeats (CRISPR) region, and the other two had sequences that were similar to the Japanese isolate MAFF311018 and the Philippines isolate PXO563, respectively. The genomes of two Taiwanese isolates with unique CRISPR sequence features, XF89b and XM9, were further completely sequenced. Comparison of the genome sequences suggested that XF89b is phylogenetically close to MAFF311018, and XM9 is close to PXO563. Here, documentation of the diversity of groups of *Xoo* in Taiwan provides evidence of the populations from different sources and hitherto missing information regarding distribution of *Xoo* populations in East Asia.

## Introduction

*Xanthomonas* is a large genus in γ-proteobacteria and causes diseases on more than 400 plant species. Two pathovars within the same species, *Xanthomonas oryzae* pv. *oryzae* (*Xoo*) and *Xanthomonas oryzae* pv. *oryzicola* (*Xoc*), are the most prevalent pathogens that cause diseases in rice around the world. They have a high degree of tissue specificity as well as the host-plant specificity^1^. *Xoo* and *Xoc* are distinguished by the tissue types they colonize in rice leaves. *Xoo* colonizes vascular tissues and causes leaf blight disease, whereas *Xoc* invades the mesophyll parenchyma tissues to cause leaf streak disease. *Xoo* can cause disease in both major rice sub-species, *O*. *sativa* subsp. *japonica* and *O*. *sativa* subsp. *indica*. However, *Xoc* cannot form severe disease symptoms in *japonica* rice^2^. *Xoo* has a larger host range and causes more serious problems in rice than *Xoc* in Asia, especially in East and East-Northern Asia^2^. *Xoo* can be further classified into races based on ability to infect different rice cultivars. To date, over 30 races of *Xoo* have been discovered all over the world^3–5^, and the rice blight disease can cause the yield losses up to 70% under environmental conditions favorable to *Xoo* infection^6^. Cultivation of *Xoo*-resistant rice cultivars is the most effective way of dealing with the blight disease, which would require the establishment of the distribution patterns of clonal *Xoo* populations in the rice growing regions.

The rice blight disease was first reported in Taiwan in 1951^7^, but occurred infrequently in the fields before 1970s^8^. However, after an outburst of rice bacterial blight impacted the local rice cultivation system in 1980s, this disease became a serious cultivation issue in Taiwan^8^. Although the race identification of local *Xoo* isolates has been reported since then, the isolates and rice varieties used for the classification are limited^8–11^ and a better understanding of *Xoo* population dynamics in Taiwan is needed. Moreover, the *Xoo* populations in Taiwan cannot be compared with populations in the neighboring regions, because (i) the rice hosts selected for race identification are inconsistent in the local studies^8–11^, (ii) there were insufficient genetic markers for the identification of populations^12^, and (iii) the population analyses of Asian *Xoo* in prior studies did not include any samples collected in Taiwan^13,14^.

Rice grown in Taiwan is composed of many different varieties^15^, with the temperate japonica *O*. *sativa* being the major cultivar. This type of rice in Taiwan was originally adopted from Japan in the early 20^th^ Century and naturalized through breeding programs. However, the major variety of *O*. *sativa* in Taiwan before the 20^th^ century was the indica subspecies from China. With rice varieties from two different origins being cultivated together, *Xoo* in Taiwan may have undergone a unique evolutionary path. In addition to the different pathogenicity of *Xoo* races toward rice cultivars, the major race of *Xoo* may also be different both in geographic regions and time periods. Since *Xoo* is a rapid evolving pathogen^16^, the selection of cultivated rice varieties may facilitate the race shift or result in the emergence of new races. Indeed, a prior study showed that the shifting of the major race over time in the Philippines might be caused by a dramatic change in the host genotypes^17^. Accordingly, the local rice cultivar-driven changes of *Xoo* populations increases the difficulties in preventing this rice disease, and further emphasizes the necessity for local surveys of *Xoo* populations. By comparing local populations to *Xoo* populations in neighboring regions, it would be possible to delineate the relationships among them, and further focus on the fast-evolving pathogenicity-related genes for breeding of the blight-resistant rice.

Among the available genetic analyses, the insertion sequence *IS1112*, a relatively high copy-number repetitive element in the *Xoo* genome^18^, and the repetitive extragenic palindromic (REP) element have been used to characterize the isolates in Taiwan^12^. However, the groupings based on these two methods are not sufficient to classify the local isolates, and these studies do not provide comparative information among local populations and populations in the neighboring regions^12^. To address these issues, we used a robust genetic classification platform to establish the distribution patterns of *Xoo* clonal populations in Taiwan. We further used a genetically hereditable sequence, clustered regularly interspaced short palindromic repeats (CRISPR) and genomic sequencing of representative *Xoo* isolates to study population dynamics of *Xoo* in Taiwan and neighboring regions. Our analyses reveal the complexity of rice cultivation history and indicate that *Xoo* populations in Taiwan may have evolved from multi-sources and undergone a unique evolutionary path.

## Results and Discussion

### *Xoo* isolates in Taiwan have five distinct clonal populations

*Xoo* isolates in Asia have been suggested to have evolved into five modern genetic populations by RFLP or variable-number tandem-repeat (VNTR) analysis^13,14^. Nonetheless, isolates in Taiwan were not included in those studies. We used RAPD primers employed in prior studies^19^ to analyze 51 Taiwanese isolates collected from 1986 to 2010. However, 38 of these primers had low resolutions in the isolates, and only 11 of them could be used for grouping. These results indicate that *Xoo* isolates in Taiwan are genetically close to each other. However, from these 11 RAPD markers, we could still separate the 51 isolates into five different clonal populations named R1 to R5 (Supplementary Fig. S1).

We next used RFLP to reconfirm the grouping of *Xoo* isolates in Taiwan by RAPD. This method has been widely used to assist race identification as well as the molecular classification^20,21^. Here, we used the *avrXa7* (one of the TALE) repeat region as a probe to classify the pattern of *Xoo* isolates generated from RFLP (Supplementary Fig. S2a). The grouping of TALE patterns resulted in four major groups (T1 to T4) and two outgroup isolates, XO4a (T5) and XM9 (T6) (Supplementary Fig. S2b). The RAPD and RFLP analyses generated similar groupings, except the T2 group of the RFLP analysis was split into R2 and R5 groups in the RAPD classification.

**Figure 1:**
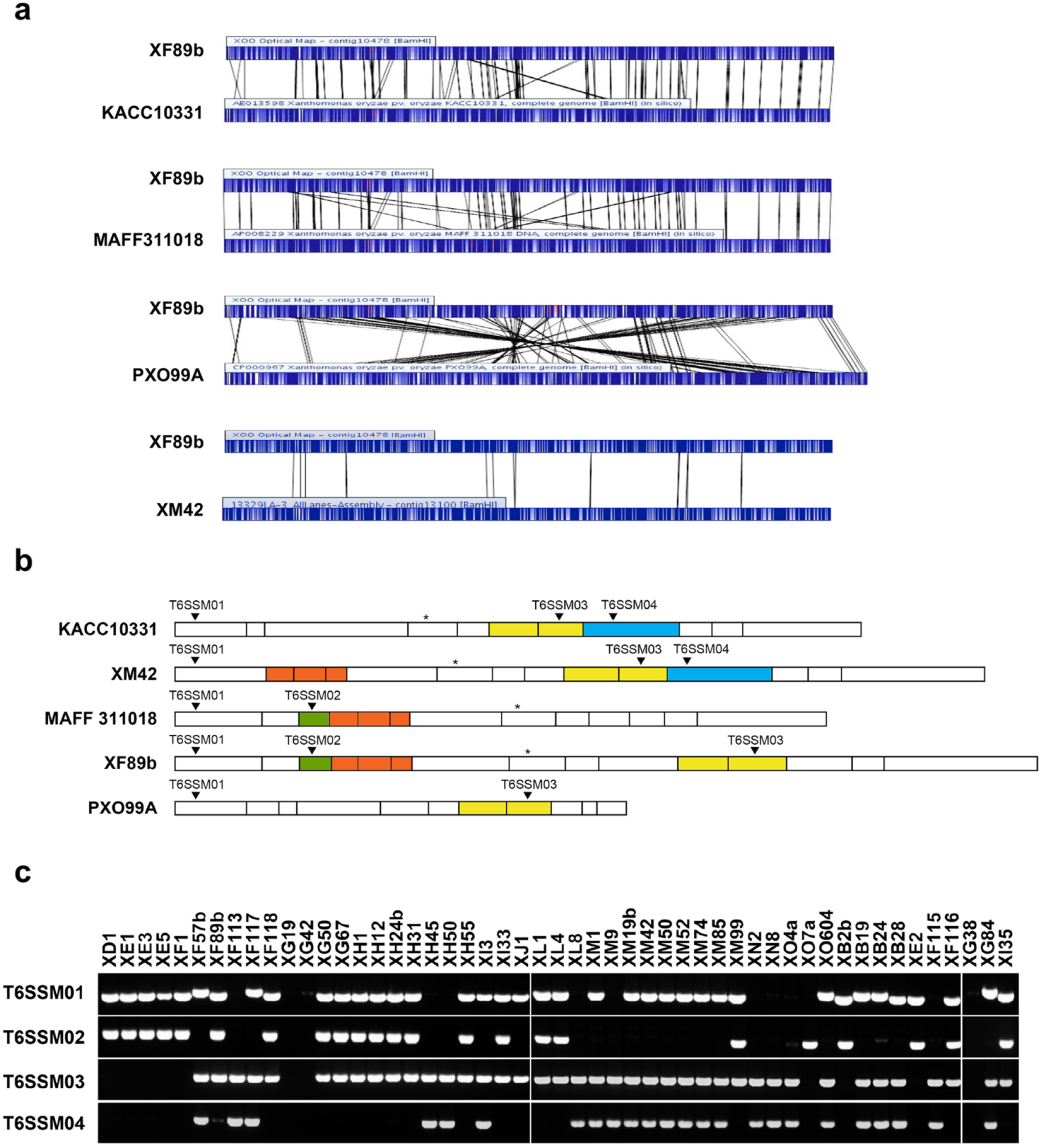
T6SS-II region and marker polymorphism in Taiwanese isolates. (**a**) Genome restriction maps of XF89b, XM42, KACC10331, MAFF311018, and PXO99A. The maps were generated using the optical mapping technique with a restriction enzyme, BamHI. Black lines indicate similar restriction patterns between two isolates. (**b**) Restriction maps in the T6SS-II region among XM42, XF89b, MAFF311018, KACC10331, and PXO99A. The interval lines indicate the cutting site of BamHI. The star indicates the location of one of the core genes, *Hcp2*, in the T6SS-II island. Colored regions in the maps indicate the relative similar patterns between each isolate. (**c**) Four T6SS-II marker primers were designed based on the presence or absence of particular sequence regions between isolates in (**a**). The predicted amplification region of the markers named T6SSM01 to T6SSM04 were marked in (**b**) in each isolate, and the amplification results by PCR were performed in 51 Taiwanese isolates.

**Figure 2:**
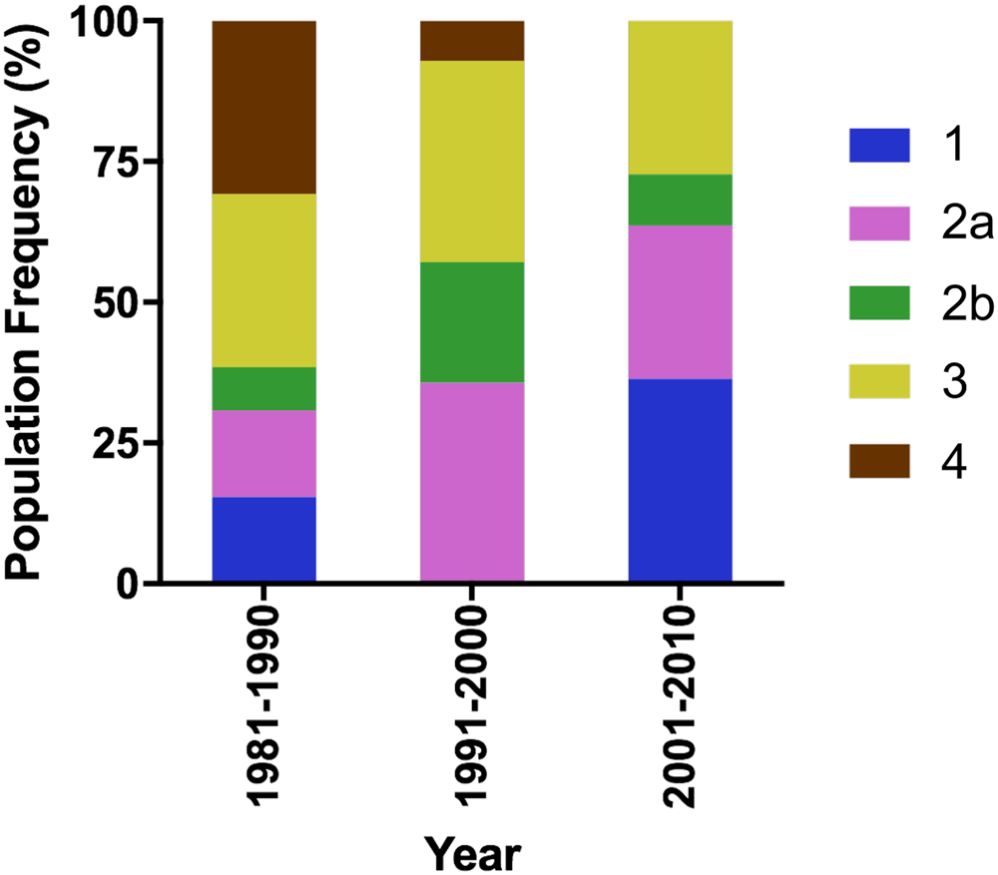
*Xoo* clonal populations in Taiwan over a 30-year collection period (1981 to 2010). Frequency of discovery rate in each clonal population during three survey periods. Each color represents a distinct clonal population in Taiwan. The number of *Xoo* entries in the following periods are: 1981–1990 = 26, 1991–2000 = 14, 2001–2010 = 11.

In order to generate novel and reliable markers for fast identification and confirmation of the *Xoo* isolates in Taiwan, we introduced a third method, i.e., searching for indel regions of the reference isolates and different signatures of restriction maps between two local isolates, XF89b and XM42. Due to their high virulence, these two isolates were used to select for *Xoo*-resistant rice cultivars in Taiwan over 30 years. We generated genome restriction maps of XF89b and XM42 using an optical mapping technique (Fig. 1a) and compared them to the maps of KACC10331, MAFF311018, and PXO99A, which were generated by *in silico* genome restriction mapping from their respective genomic sequences^16,22,23^. We found that the region within and near T6SS-II is highly variable among these isolates (Fig. 1b), which is similar to the study on *Pantoea ananatis*^24^. Hence, we designed four molecular markers for the grouping. These four markers could generate eight different patterns (S1–S8) in all the Taiwanese isolates and thus increased the classification accuracy (Figs 1c and S3). In this marker identification assay, we observed that most of the isolates showed consistent classification as in the RAPD analysis. Only five isolates had different marker patterns and thus were grouped into additional classes (S6–S8) (Supplementary Table S1). By analyzing the binary data generated from these three grouping strategies (Supplementary Figs S1a and S2b, Table S1), these 51 isolates were classified into five major groups. Two groups were evolutionarily closer to each other by the neutral marker RAPD analysis, so we designated these five groups as G1, G2a, G2b, G3, and G4 (see Supplementary Fig. S1, Table S1). Population surveys showed that from 1981 to 2010 the G1 population increased and the G4 population decreased, whereas G2a and G3 populations remained relatively steady (Fig. 2). G2a and G3 constitute the major clonal populations in Taiwan, and they accounted for 54.9% (28/51) of the collected samples (Fig. 2). All five groups showed a geographically related clonal distribution (Supplementary Fig. S4). G1 and G2b were mainly collected in the northern part of Taiwan, and G4 was mostly discovered in the southern part, whereas G2a and G3 could be found from central to southern Taiwan.

### CRISPR spacer sequences reveal the relationship among *Xoo* clonal populations from different geographic regions

**Figure 3:**
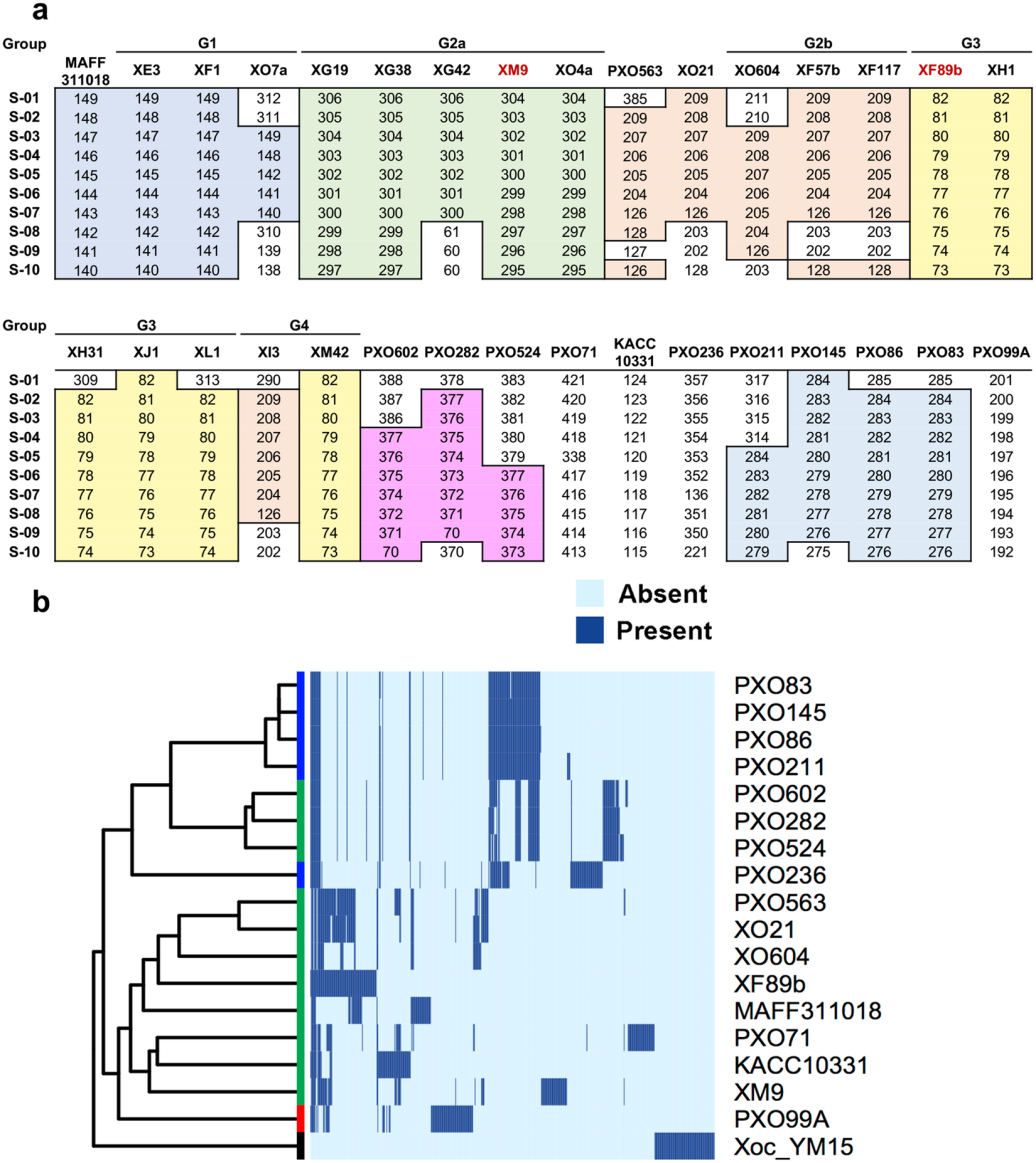
Alignment of CRISPR spacers from the *Xanthomonas oryzae* pv. *oryzae* (*Xoo*) genomic sequences. (**a**) Comparison of the last 10 spacer sequences beginning from LDR. The same color block indicates the same or similar (<2 nucleotides mismatch) spacers shared in the group. The first column represents the order of the 1^st^ (S-01) to the 10^th^ (S-10) of the last 10 spacers in the CRISPR region. The number indicates the sequence ID in the spacer library (Supplementary Table S2). (**b**) Alignment of entire CRISPR spacers in all fully sequenced *Xoo* isolates. *Xoc* YM15 is the only *Xoc* isolate containing CRISPR system, and this isolate is used as an outgroup in the analysis. The colored bar at the end of the phylogenetic tree represents the groups defined from genome SNP analysis (Fig. 4a).

To further characterize these clonal populations, we used the CRISPR region to establish the relationship among isolates from Taiwan, the Philippines (PXOs), Japan (MAFF311018) and Korea (KACC10331). The CRISPR region contains sequences derived from lytic or filamentous bacteriophages, or conjugative plasmids (also known as spacers) adjacent to its leading sequence region (LDR)^25^. Therefore, the earlier acquired sequences become more distanced from LDR and provide evolutionary information through which the relationship among bacterial isolates can be identified^16,25^. The CRISPR regions of nineteen isolates from each Taiwanese clonal population were randomly chosen, and the 10 spacers near the LDR were sequenced or obtained from a prior study^26^. As expected, the spacer sequences and order among Taiwanese isolates were similar within the same group but varied between groups (Fig. 3a, Supplementary Table S2). Interestingly, we found that G1 isolates had spacer sequences and order that is almost identical to the Japanese isolate MAFF311018, suggesting that G1 has a close phylogenetic relationship with this Japanese isolate. In contrast, G2b shared similar spacer sequences and order with PXO563 (Philippine race 10) with only two differences being found within these 10 spacers. However, G2a and G3 had sequences and order within this region that were distinct from both the Japanese and Philippine isolates, as well as other known CRISPR sequences in *Xoo*. Two G4 isolates XI3 and XM42 had different spacers, with XI3 being similar to G2b and XM42 similar to G3, suggesting that G4 group is a hybrid group, with its CRISPR regions inherited from either G2b or G3.

Interestingly, phylogenetic analysis using spacer sequences of the entire CRISPR region revealed that among the Philippine *Xoo* subgroups^17^, with the exception of PXO99A, PXO71 and PXO563, all the isolates were grouped together and separated from *Xoo* collected from Japan (MAFF311018), Korea (KACC10331), and Taiwan (XO21, XO604, XF89b and XM9) (Fig. 3b, Supplementary Table S3), which is consistent with prior studies^17^. The Philippine race 6 (PXO99A) has been considered as a foreign clonal population in the Philippines based on the molecular features, and the Philippine race 10 (PXO563) is an emerging clonal population in that area^17^. In our analysis, the G2b group of *Xoo* isolates, which were highly similar to PXO563 in CRISPR spacers, were first collected during the 1981 to 1990 period and continuously observed in the next two survey periods (Fig. 2), whereas the Philippine race 10 (PXO563) had only been collected in 1998 to 2002 period and therefore only accounted for a small proportion of total samples collected in that period in the Philippines^17^. Considering the appearance time and proportion of the Philippine race 10 (PXO563) in the Philippines and its close relationship with the G2b group in Taiwan, the Philippine race 10 (PXO563) might have spread from Taiwan or nearby countries to the Philippines. The Philippine race 4 (PXO71) might also be a foreign clonal population, because it lacked the typical patterns of the Philippine isolates in CRISPR analysis (Fig. 3b). However, the Philippine race 4 (PXO71) accounts for only a small portion of the collected samples^17^ and does not share any last 10 spacer sequences with the Taiwanese populations (Fig. 3a). Hence, the origin of this race needs to be further investigated.

Our CRISPR spacer analysis also revealed that XF89b, XO604 and XO21^26^ were clustered together in the same clade with the Japanese isolate MAFF311018, but each isolate also had unique spacers (Fig. 3a,b). However, RAPD, RFLP, and T6SS-II marker assays put XF89b in the G3 group and XO604 in the G2b group, and they are distinct from G1 group. In contrast, the XM9 (G2a) population has a closer relationship with KACC10331 and PXO71 than other populations. Taken together, these results showed that the distribution of clonal populations could be reflected by the CRISPR spacer sequence, and the grouping results from CRISPR spacers were in general similar to our other molecular analyses.

Due to intense breeding of pathogen-resistant rice cultivars, *Xoo* has to evolve fast to overcome new resistant traits, and the dominant population may shift from one race to another locally and temporally because of the different virulence toward the rice cultivars. In the Philippines, population shift was observed across a 40-year-period, and three dominant populations, PXO602(3c), PXO282(1) and PXO524(9b) accounted for over 90% of *Xoo* populations across the latest two survey periods from 2003 to 2012^17^. The genomic analysis^17^ and CRISPR analyses suggest that these three major races share a highly similar genetic background and were recently separated from each other (Fig. 3b). Unlike the situation in the Philippines, the population shift in Taiwan is not obvious by the molecular classification. At least two populations, G2a and G3 have dominated the *Xoo* populations in Taiwan over a 30-year-period (Figs 2 and 3a). In addition, the phylogenetic analyses indicated that *Xoo* populations in Taiwan evolved independently from the major Philippine populations and were closer to the Japanese and foreign Philippine isolates, MAFF311018 and PXO563 (Fig. 3b).

### XF89b and XM9 belong to two different groups within the Xoo-A clade

**Figure 4:**
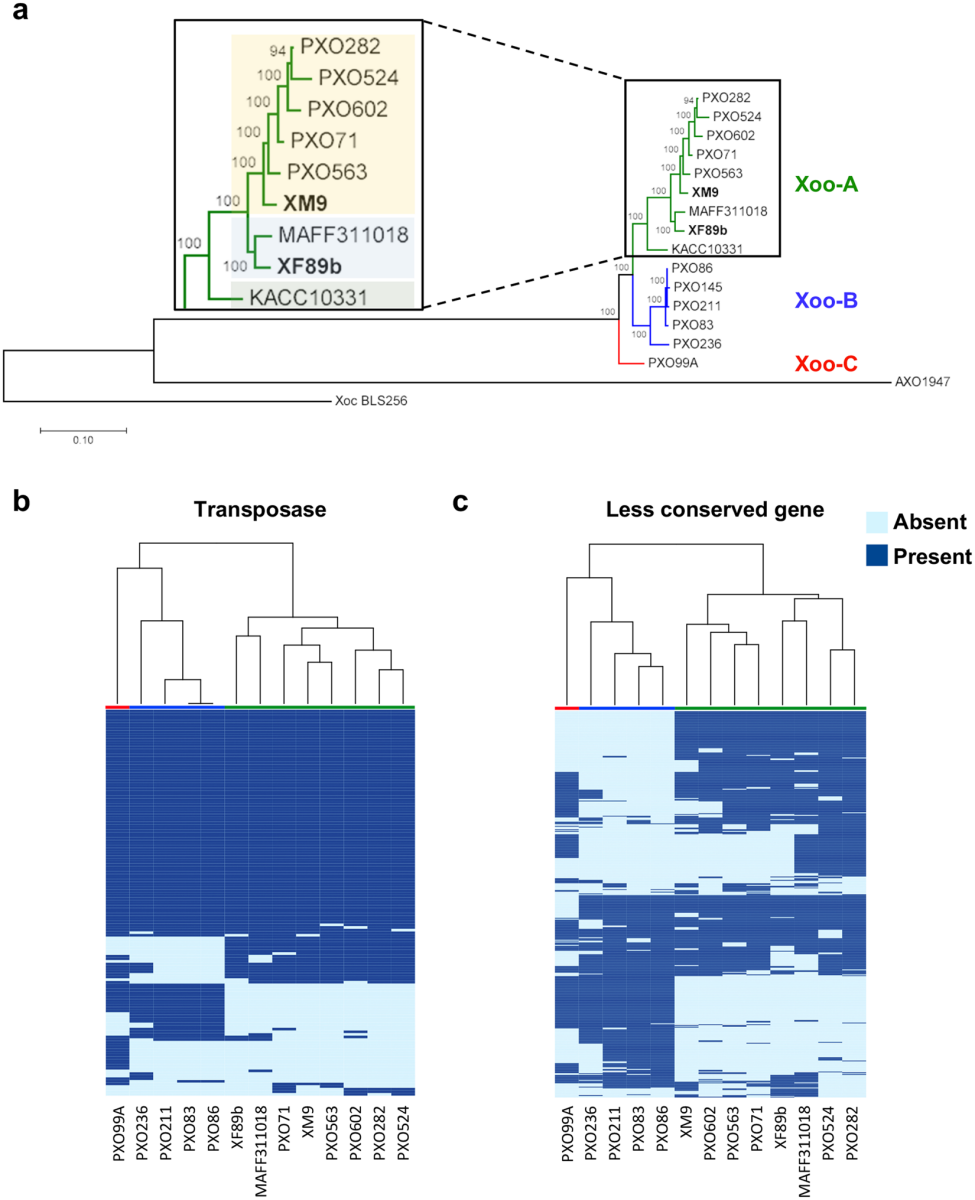
Phylogenetic relationships of *Xanthomonas oryzae* pv. *oryzae* (*Xoo*) isolates. (**a**) Maximum likelihood phylogenetic tree of sequenced *Xoo* isolates is based on 86,919 concatenated core SNPs. Xoo-A, Xoo-B, and Xoo-C groups are indicated by the green line, blue line and red line, respectively. In Xoo-A, three different background colors represent distinct subgroups. African *Xoo* isolate, AXO1947, and *Xoc* BLS256 were used as outgroups. (**b**,**c**) A heatmap of hierarchical clustering is based on 148 transposases between *Xoo* isolates (**b**) and 751 less conserved genes between *Xoo* isolates (**c**), and the colored bar represents the groups defined from genome SNP analysis.

Since G2a and G3 have unique CRISPR spacers that have not been reported before, we chose the isolate XM9 from G2a, which has a unique TALE signature, and XF89b from G3, which has a typical TALE pattern (Supplementary Fig. S2a), for genome sequencing. The whole genome sequences indicate that XM9 and XF89b had similar genome structures with KACC10331, MAFF311018, and those of the Philippine *Xoo* group PX-A^17^ (Supplementary Fig. S5). A SNP phylogenetic tree was further created by *Xoo* genome sequences, and these *Xoo* isolates were categorized into three major clades, which were named as Xoo-A, Xoo-B, and Xoo-C (Fig. 4a). The tree showed that KACC10331, MAFF311018, XF89b and XM9 were clustered into Xoo-A clade, while the isolates from the Philippines^17^ were grouped into Xoo-A, Xoo-B and Xoo-C, respectively (Fig. 4a). Unlike the CRISPR region analysis (Fig. 3b), PXO282, PXO524, and PXO602 were separated from other Philippine isolates and clustered to the Xoo-A clade (Fig. 4a). The CRISPR spacers evolve by either stepwise acquisition or recombinational loss or duplication of several spacer-repeat units^25^. Although the stepwise acquisition is the major evolutionary event of CRISPR elicited by environmental challenges, the rare events of spacer losses or duplications contribute to the footprint of spacers in the CRISPR region. They also affect the distance of the phylogenetic tree analysis. Most of the Philippine *Xoo* isolates contain a similar spacer footprint that is different from isolates collected from other areas and thus grouped together. Also, CRISPR in bacteria serves as an immune system^27^. Under frequent environmental challenges, the CRISPR region of *Xoo* might be under a stronger selection force than most of the other functional regions and obtain or retain the same spacers in the genome. It could further explain the different grouping results between CRISPR spacer analysis and genome SNP analysis.

Within Xoo-A group, the isolates collected from different countries could be further divided into three subclades, which revealed that XF89b was clustered closely with MAFF311018 (Fig. 4a). Linkage distance analysis with bootstrap test showed that the XF89b branch had longer individual distance in the phylogenetic tree and distinct evolutionary direction from other isolates in Xoo-A, including XM9, suggesting that it was under adaptation process (Fig. 4a). Besides, in transposase and less conserved gene analyses, XF89b and XM9 belonged to different subclades of Xoo-A (Fig. 4b,c). These results suggest that there are at least two distinct major clonal populations in Taiwan. XM9 might share a more recent ancestor with Philippine Xoo-A isolates and XF89b evolved from a later common ancestor of the Japanese isolate MAFF311018.

### T6SS-II evolved rapidly in *Xoo*

**Figure 5:**
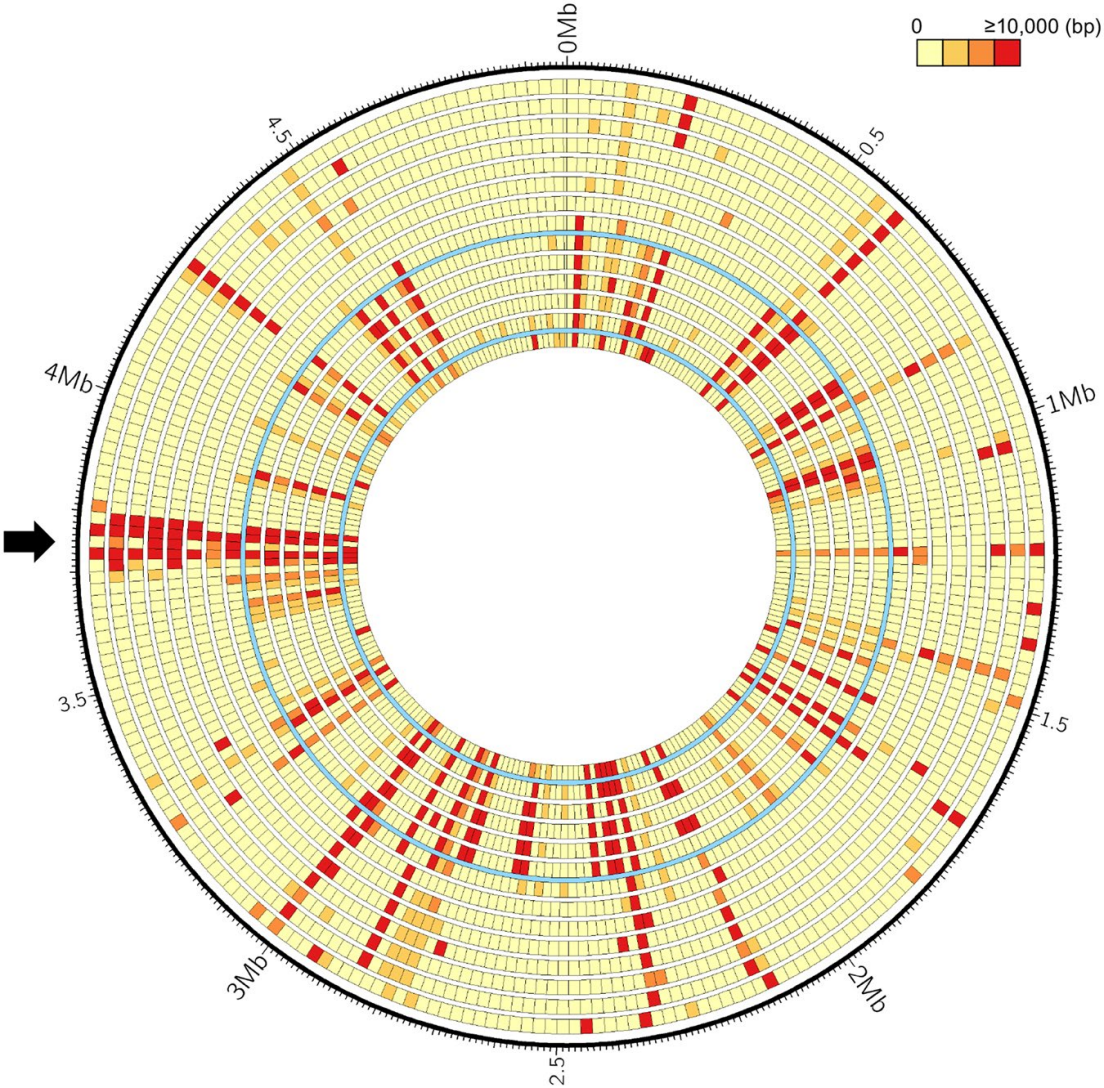
A heatmap of indel comparison in *Xanthomonas oryzae* pv. *oryzae* (*Xoo*) isolates. The window of indel map was fixed to 10 kb for each block, and the number of base-pair differences was generated from the gap detection function in MAUVE^52^. The cyan lines separate Xoo-A, Xoo-B, and Xoo-C clades of *Xoo* isolates and the arrow indicates T6SS-II region of *Xoo* isolates. The circles from outside to inside: XF89b versus PXO282, PXO524, PXO602, PXO71, PXO563, XM9, MAFF311018, KACC10331, PXO86, PXO145, PXO211, PXO83, PXO236, and PXO99A.

In bacterial species, recombination events drive evolutionary fitness and have long- term effects. However, the isolates in the Xoo-A clade share a highly similar genome structure and might have undergone a bottleneck event in their ancestry^17^. Although the recombination events were not strong enough to disrupt the linkage signal in the Xoo-A group due to strong host selection, it might still be an important source of variability. Thus, we focused on searching for the unique genes and recombination events. We found that a region containing T6SS-II and putative T6SS-related genes were variable in their sequences in the indel map (Fig. 5). T6SS-II of *Xoo* was considered to contain a large number of T6SS-unrelated ORFs interspersed with T6SS-related genes^28^. After annotating the genes in this region, many hypothetical genes were found after the *VgrG*-like gene, and each *VgrG*-like gene was considered as the first gene in the putative operons without intergenic non-coding region between genes (Supplementary Fig. S6). *VgrG* operon has been shown to contain toxin and anti-toxin pairs for bacterial competition and preventing suicide^29,30^. Therefore, these hypothetical genes might be T6SS-related genes and under T6SS-II regulation. Notably, none of the genes annotated as a unique gene were found in this region, and the genome structure is different from each other among Xoo-A isolates. Taken together, these results indicate that T6SS-II region is not only variable between the Xoo-A, Xoo-B and Xoo-C groups, but frequently rearranged even within the Xoo-A group, in which the isolates had similar genomic backgrounds. This finding implies that the evolutionary rate might be high in this region. These results also suggest that T6SS-II plays an important role in either host-pathogen interaction or the survival of *Xoo* in the environment.

### XF89b and XM9 carry different sets of T3SS effectors

**Figure 6:**
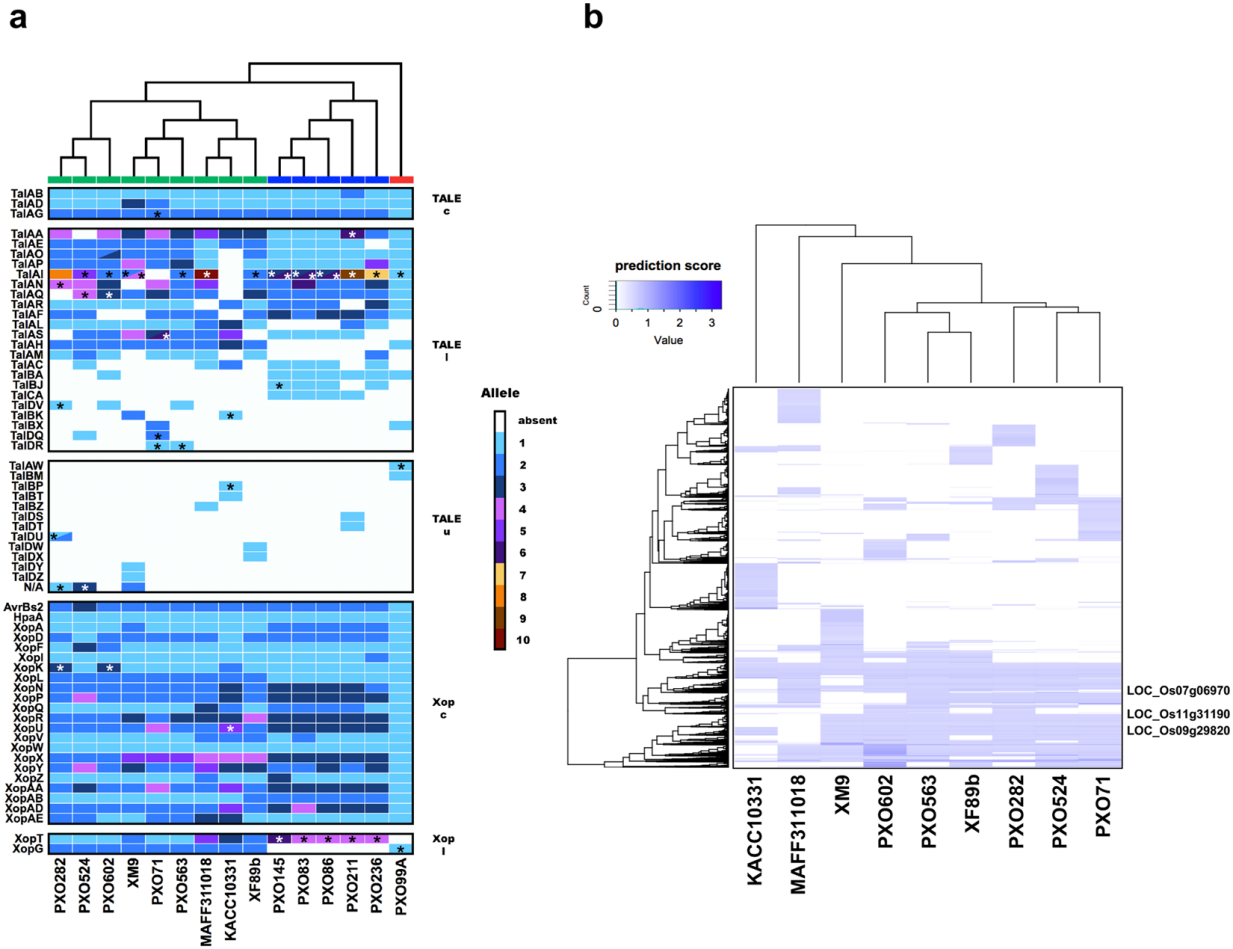
Genome comparison of *Xanthomonas oryzae* pv. *oryzae* isolates. (**a**) Comparison of type III secretion system effectors in analyzed *Xoo* isolates with genome sequences. Different allele types of effectors were grouped and colored. The color scale indicates types of alleles present in each class of effectors. N/A represent TALEs that were not assigned to any class in AnnoTALE^33^. Different colors in the N/A group do not share similar RVD compositions. Stars represent truncated TALEs/interfering TALEs or pseudo-Xop genes. The color-bar at the top of the heatmap indicates the classification based on genome SNPs. On the right y axis: c; core gene, l; less conserved gene, and u; unique gene. (**b**) TALE target prediction of Xoo-A group of *Xoo* isolates. A recognition possibility data of 200 predicted target genes of the rice host in each TALE were generated using TALVEZ^55^, and an *Oryza sativa* genome, MSU7, was used as the template for target prediction. The top one target of the list was normalized to 1, and values for different TALEs that targeted to the same gene were calculated as a sum of the normalized values in each TALE. The known TALE-targeted gene IDs were shown at the right side of the heatmap. The gene IDs and recognition scores are shown in Supplementary Tables S6 and S7.

T3SS effectors, including TALEs and Xops, are one of the most important classes of effectors in *Xanthomonas* pathogens that are related to the overall pathogenicity of each *Xoo* isolate^2,17,31^. The TALEs has a conserved repetitive region in the middle of their coding sequences, and the variations at 12^th^ and 13^th^ amino acid of each repeat, so called repeat variable diresidue (RVD), determine the binding specificity of the nucleotide^32^. The binding targets of TALEs play important roles in rice resistance towards *Xoo*^2^. Also, Xops can determine the virulence of *Xoo*^31^. Therefore, the classification of these effectors might provide additional information to clarify the differences between each *Xoo* isolate. Here, we used all the TALEs including previously classified TALEs^33^ and Xops^17^ to group all the sequenced *Xoo* isolates (Fig. 6a, Supplementary Tables S4 and S5). The grouping pattern was similar to that of the genome SNP classification (Fig. 4a). As expected, XF89b shared large numbers of T3SS genes with MAFF311018, as they are the closest clones based on the genome sequences. However, XM9 has quite different T3SS gene sequences, from its closest reference isolates PXO71, PXO563 and local isolate XF89b. This is consistent with the observation that XM9 contains a unique TALE pattern, which is distinct from other *Xoo* isolates in RFLP analysis (Supplementary Fig. S2). In the genome TALE distribution analysis, we found that TALE islands in the genome are highly conserved within each *Xoo* group (Supplementary Fig. S7), and rearrangements of TALEs between each island are usually accompanied by genome retro-rearrangements in the nearby regions (Supplementary Fig. S5).

XF89b had a similar TALE distribution to its closest isolate MAFF311018. The fifth TALE island of both XF89b and MAFF311018 contains a single pseudo-TALE with a similar repeat region, but XM9 and its closest isolates, PXO563 and PXO71, have two pseudo-TALEs in the region, one of which is similar to the pseudo-TALE in XF89b and MAFF311018 (Supplementary Fig. S7, Table S5). Moreover, XF89b and MAFF311018 shared the same type of TALE gene, *Tal2b*_XF89b_ (allele type 2 of TalAB), whereas XM9, PXO563 and PXO71 shared the other type of TALE gene, *Tal2b*_XM9_ (allele type 1 of TalAB) in the corresponding position (Supplementary Fig. S7). In order to investigate whether there are some TALEs that are uniquely evolved in Taiwan, we used TALE sequences of *Xoo* isolates from other regions and Taiwanese isolates to generate a DisTAL tree using each repeat of TALEs as a unit and transform it into a coded repeat^34^. The pair-wise alignment was then applied to construct the tree. From the DisTAL tree, we found that XF89b and XM9 shared five identical TALE repeat arrays (TalAA, TalAE, TalAH, TalAO and TalAR). Nevertheless, other TALEs were in different sub-roots (TalAB, TalAD, TalAP and TalAQ). XF89b and XM9 also share two identical RVDs of the TALEs in the AnnoTALE^33^ classes (TalAG and TalAL) but have short distance in DisTAL tree. This is caused by the amino acid variations in the repeat arrays other than RVDs. Also, these two isolates had eight TALEs classified into different AnnoTALE classes and sub-roots (see Supplementary Fig. S8, Table S4). These results suggest an early divergence occurred between XF89b and XM9. Interestingly, the DisTAL tree showed that one of the TALEs in XM9, *Tal3a*_XM9_, was an outgroup of all the analyzed TALEs. It has a remarkably short sequence with a 3.5 repeat array and a complete TALE sequence without disruption. Indeed, this short repeat array was also shown in RFLP analysis (Supplementary Fig. S2). This small fragment was not unique in XM9, 13 (27%) other Taiwanese isolates also contained this or similar-sized fragment. Over a quarter of the collected samples have the short central repeats, implying that it is not a recent mutated form. This TALE is believed to be too short to bind DNA^32^. Recently, a study on Xo1 suggests that TALEs with short central repeats (at least 3.5 repeats) are required to trigger the rice resistance. However, studies on truncated TALEs (truncTALEs) and/or interfering TALEs (iTALEs) reveal that truncTALEs/iTALEs can mimic the TALE structure and overcome the Xo1- and Xa1-mediated resistance^35,36^. Accordingly, it remains to be clarified whether these small TALEs function as iTALEs and are recognized by Xa1 and Xo1.

Two R genes in rice, *Xa27* and *Xa7* that are effective against specific *Xoo* isolates, have been well studied^37–40^. Although XF89b and MAFF311018 are the closest isolates in genome SNP analysis, only XF89b, but not MAFF311018, seems to have escaped the *Xa27* gene activation trap. This is probably because XF89b has a TALE in the allele type 2 of TalAO class, which is distinct from *AvrXa27* (allele type 1 of TalAO) in MAFF311018^17^ (see Supplementary Table S5) and that the activation of the *Xa27* gene in rice is initiated by the binding of *avrXa27* on its promoter region^41^. This phenomenon was also observed in XM9, suggesting that *Xa27* is not an efficient resistance gene against Taiwanese isolates. In addition, XF89b and XM9 do not have an *AvrXa7* (TalAC class) or an *AvrXa7* homolog (TalDV class) (see Supplementary Table S5). The TALEs in XF89b and XM9 that are closest to *AvrXa7* are *Tal6c*_XF89b_ (TalDW1) and *Tal6c*_XM9_ (TalDZ1) (Supplementary Fig. S8, Table S5). However, these two TALEs have longer TALE repeat arrays and different dipeptide compositions from other reference TALEs. They are similar to each other but still have minor variations in dipeptide compositions. This observation suggested that *Tal6c*_XF89b_ and its related TALEs in Taiwanese isolates might have evolved from *AvrXa7* under higher selection pressure.

### XM9 and XF89b differentially regulate TALE-targeted rice genes in TNG67

Prior studies showed that many TALEs have the ability to cause disease lesion by up-regulating expression of certain host genes^17^. Thus, TALE target prediction also provides insight on how rice cultivars are affected by *Xoo* at the molecular level. Here, we compared the predicted rice gene targets potentially recognized by TALEs in the Xoo-A group (Fig. 6b, Supplementary Tables S6 and S7).

**Figure 7:**
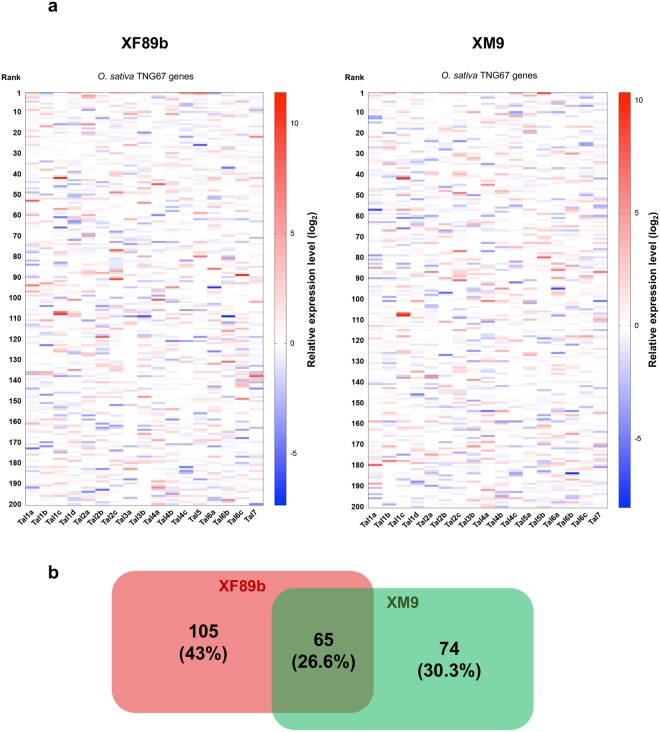
Transcriptome profiles of predicted TALE-targeted rice genes on *O*. *sativa* TNG67. (**a**) The up-regulated and down-regulated genes of *O*. *sativa* TNG67 were profiled after 7 days of *Xoo* XF89b and XM9 inoculation compared to mock-inoculated rice samples, and the relative expression levels of the top 200 TALE-targeted genes predicted by TALVEZ^55^ are listed. The relative expression levels were calculated from infected samples divided by mock-treated samples. Every column represents a different gene list which was predicted as the putative targets of individual TALEs. TAL3a_XM9_ only had three DNA binding residues, and its targets were excluded from the prediction lists. (**b**) The Venn diagram of up-regulated genes (≥2-fold changes) in the TALE-targeted prediction lists between XF89b and XM9. A total of 244 putative TALE targets in XF89b and XM9 were included in the comparison.

XM9 and XF89b had distinct lists of predicted TALE-targeted genes (Fig. 6b, Supplementary Table S7). Therefore, they might regulate different sets of rice genes during colonization. To evaluate the up-regulated genes at 7 day-post-inoculation (dpi) with *Xoo* isolates compared to mock-inoculated rice leaves in the predicted TALE-targeting lists, we used RNA-seq to examine the gene expression profiles of the TALE-targeted genes after inoculation of XM9 and XF89b (Supplementary Table S8). The heatmap of expression levels of the putative TALE-targeted genes showed that some of the genes were highly expressed after *Xoo* infection and ranked as the top 10 genes in the lists, but some of them did not have high rankings (Fig. 7a). This indicates that the predicted lists combined with transcriptome data may enhance the chance of finding the real targets of TALEs. Moreover, when we compared the putative TALE-targeted gene lists in XM9 and XF89b, only 26.6% of genes were simultaneously up-regulated. Many other genes were either unique in the strain-specific putative TALE-targeted gene lists or only up-regulated by one of these two isolates (Fig. 7b).

Next, we focused on the two TALEs, *Tal6c*_XF89b_ (TalDW1) and *Tal6c*_XM9_ (TalDZ1), because these two TALEs were close but different from the *AvrXa7* (TalAC) or the *AvrXa7* homolog (TalDV). The predicted targets for these two TALEs were different. *Tal6c*_XM9_ had the highest prediction score to target *Os11N3* (Supplementary Table S6), which is also a direct target of *AvrXa7*^42^. Nevertheless, this rice gene was only up-regulated 3.2-fold (Supplementary Tables S8 and S9). In addition, several other rice genes predicted as *Tal6c*_XM9_ targets had higher expression levels than *Os11N3*, but most of them have unknown functions. In contrast, *Tal6c*_XF89b_ is predicted to target *LOC_Os04g58860* (Supplementary Table S6). However, this rice gene was not induced under *Xoo* XF89b infection (Supplementary Table S9). Also, *Os11N3* was only up-regulated 5.7-fold (Supplementary Table S9). Instead, several other unknown proteins, such as *LOC_Os05g48840* and *LOC_Os10g06000*, were up-regulated to 2708-fold and 107-fold, respectively (Supplementary Table S9). We hypothesize that the variations in *AvrXa7* and *AvrXa7* homologs were caused by the selection pressure of *Xa7*, since the TALE and its homologs showed a high correlation with IRBB7 rice isogenic line containing a single R gene, *Xa7*^17^.

In the other cases, *Tal1a*_XF89b_ and *Tal1a*_XM9_ were in the TalAP class (Supplementary Table S5). *Tal1a*_XF89b_ presented as the major form (allele type 1 of TalAP) in the class, and *Tal1a*_XM9_ had some variations that were not identical to other TalAP members. They share the same target gene, *OsHEN1*, in the prediction list (Supplementary Table S6). *OsHEN1* is probably a target of allele type 1 of TalAP TALEs^43^. However, the variation form of TalAP, *Tal1a*_XM9_, failed to activate the transcription of this gene, since it was only up-regulated by 1.7-fold under XM9 infection (Supplementary Table S9). On the contrary, *OsHEN1* was up-regulated 6-fold under XF89b infection (Supplementary Table S9).

Moreover, XM9 also showed many variations in TALE repeat arrays and a different predicted target list as well as the transcriptional levels of the targeted rice genes from other TALEs within the AnnoTALE classes. This suggests that XM9 might be under a strong selection pressure to escape the gene activation traps, or it has evolved a new ability to target other host genes. Taken together, XF89b and XM9 have similar TALE island distribution in the genome as the isolates of Xoo-A clade, but they only share a few types of TALEs. In addition, they only share about one-fourth of TALE targets that can be induced by the inoculation of both isolates. Therefore, further investigation in recognition of these TALEs will be required to better characterize the effects of changes in the RVDs toward rice hosts.

### Different *Xoo* clonal populations regulate rice genes distinctively

Besides the regulations of TALE-targeted host genes, different clonal populations may also regulate different sets of host genes to overcome the obstacles from various host genetic backgrounds during colonization. From the molecular classification of Taiwanese *Xoo* isolates, we observed that G2a, and G3 dominate the populations, and G1 was increasing in the last survey period. To understand whether these three populations regulate different sets of the host genes, we chose representative isolates from these three populations, XE3 (G1), XM9 (G2a) and XF89b (G3), for transcriptomic analyses after the inoculation of local temperate japonica rice, TNG67. XE3 had exactly the same sequence and order in the last 10 CRISPR spacers with MAFF311018. Also, it showed similar TALE patterns in Southern blot assay as well as the indel marker assay with MAFF311018 (Fig. 1a,b).

**Figure 8:**
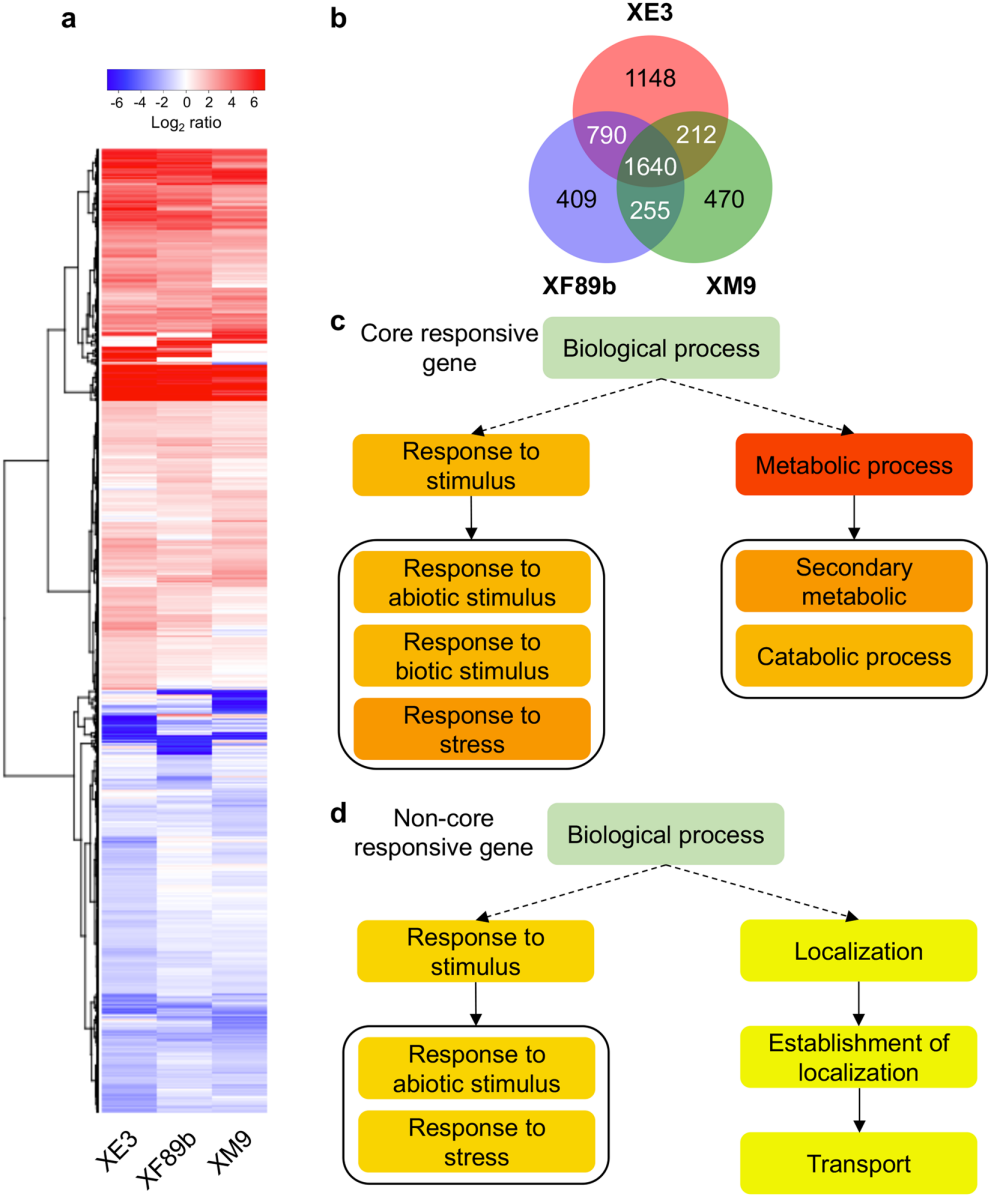
Transcriptome profiles of differentially expressed genes in TNG67 after inoculation of *Xoo* isolates. (**a**) A heatmap of differentially expressed rice genes after inoculation of *Xoo* XE3, XF89b, and XM9. The rice samples were collected at 7 days-post-inoculation (dpi). A total of 4924 up-regulated (≥2-fold change in at least one sample) genes, and 3856 down-regulated genes (≥2-fold change) (Supplementary Table S9) were included in the map. (**b**) The Venn diagram of up-regulated genes in all three samples. A total of 3790, 3094, and 2577 up-regulated genes (≥2-fold changes) in XE3, XM9, and XF89b were used for the diagram. (**c**) The GO enrichment of up-regulated genes that altered (≥2-fold change) in all three samples as core responsive genes. (**d**) The GO enrichment of up-regulated genes (≥2-fold changes) that were only altered in one or two samples as non-core responsive genes.

After the inoculation of TNG67 with these three isolates, we found that 4924 rice genes were up-regulated and 3856 genes were down-regulated by XE3, XM9 or XF89b (Fig. 8a, Supplementary Table S9). In the genome and molecular marker analyses, we observed that XF89b (G3) was genetically closer to MAFF311018, and XE3 (G1) was considered to belong to the same group as MAFF311018. On the other hand, XM9 (G2a) phylogenetically separated from the other four groups at the earlier time point. Hence, XE3 and XF89b may induce a more similar proportion of host genes than either XM9 and XE3 or XF89b and XE3. As expected, among the 4924 up-regulated genes in TNG67 after inoculation of *Xoo* isolates, 2430 (49.3%) of these genes were up-regulated by both XE3 and XF89b, which is more than genes up-regulated by XE3/XM9 (37.6%) or XE3/XF89b (38.5%) (Fig. 8b). Among these 4924 genes, one-third of genes were up-regulated by all inoculated isolates, and these genes might be core genes responding to *Xoo* infections. Indeed, GO enrichments showed that these genes were mainly involved in the responses to stresses and metabolic processes (Fig. 8c). Interestingly, there were still many genes that were specifically up-regulated under the infections of one or two isolates. These isolate-specific *Xoo*-induced genes were enriched in response to the stimuli and were also involved in localization and transport (Fig. 8d). As the OsSWEET11/OsSWEET14 are known as sugar transporters, and many TALEs target these transporters to create a more suitable environment for *Xoo*^44,45^, the transcript levels of transporters are crucial for the fitness of *Xoo* races. Thus, the enrichment of genes involved in transport in the non-core responsive gene list might result in different fitness of these three *Xoo* isolates toward different rice cultivars.

## Conclusions

We showed here that *Xoo* isolates in Taiwan consisted of 5 clonal populations, G1, G2a, G2b, G3 and G4. Genome SNPs and CRISPR spacer analyses suggested that G2a and G4 were evolutionarily closer to the Philippine Xoo-A group of isolates, whereas G3 and G1 were closer to the Japanese isolate MAFF311018. This phenomenon could be caused by the mixed cultivation of both indica and japonica rice originally from China and Japan, respectively. Comparison of genome indel regions and mutational analyses also revealed the possible important role of T6SS-II in *Xoo* pathogenicity. TALE analysis also revealed that XF89b and XM9 may target different genes in rice, and some of TALE genes, such as *Tal6c*_XM9_ and *Tal6c*_XF89b_, evolved faster in Taiwanese isolates than isolates in other countries. Furthermore, by genomic comparison of two local isolates with isolates from neighboring regions, we are able to depict the distribution patterns of East Asian *Xoo* populations and how they spread and adapt to local environments. These results contribute to our understanding of the population dynamics of this important rice pathogen and also provide a novel means through which *Xoo*-rice interactions may be studied in the future.

## Methods

### Bacterial isolates, plasmids, primers and isolation of genomic DNA

Fifty-one isolates of *Xanthomonas oryzae* pv. *oryzae* were collected from a randomly chosen areas in Taiwan by the Taiwan Agricultural Research Institute (Supplementary Table S10). *X*. *oryzae* pv. *oryzae* and *E*. *coli* were grown in LB medium at 28 °C for 3 days and 37 °C for 18 hours, respectively. Genomic DNA was extracted by Easy Tissue & Cell Genomic DNA Purification Kit (GMbiolab Co, Taiwan) and stored at 4 °C. Plasmids were purified by Mini Plus Plasmid DNA Extraction Kit (Viogene BioTek Co., Taiwan) and stored at 4 °C. Primers used in this study are listed in Supplementary Table S11.

### Random amplified polymorphic DNA analyses

Thirty-eight random amplified polymorphic DNA (RAPD) primers selected from Hu *et al*.^19^ were used for DNA fingerprints, and 11 of them were used for phylogenetic tree analyses. RAPD analyses was carried out by PCR amplification using 50 ng gDNA, 100 pmole RAPD primer, and 7.5 μl DNA polymerase master mix in 15 μl final volume. The 11 primers for phylogenetic tree analyses were P09, S01, Y02, AB07, AJ01, AM13, AS13, AT20, AZ16, BF04, and BG02 (Supplementary Table S11). The PCR reactions were performed in an MJ Research Thermal Cycle PCR machine. The PCR products were analyzed by 1.5% agar gel. The assays were repeated three times and only repeatable bands were selected for further analysis. The banding patterns were then coded in binary form, and the phylogenetic tree was generated using the unweighted pair-group method with arithmetic averages (UPGMA) method. Jaccard similarity coefficient was calculated by the SIMQUAL routine and the significant bootstrap probability (>70%) was obtained for 1000 repetitions.

### Southern hybridization analysis

The procedures of Southern blot were similar to a previously described method with some modifications^46^. Briefly, 3.1-kb *SphI* fragment of plasmid pZWavrXa7 were gel purified after digestion. This fragment was labeled with DIG DNA Labeling Mix (Roche) or AP Direct Labeling and Detection kit (GE) as a probe. Twenty micrograms (for DIG-labeling probe) or 500 ng (for AP-labeling probe) of genomic DNAs from *Xoo* isolates were digested with *SphI* and used for hybridization. Size markers were visualized by adding DIG/AP labels. Three independent repeats were performed in each sample and the phylogenetic tree was generated as described in RAPD analyses.

### CRISPR sequencing

The last 10 spacers of the CRISPR region were sequenced as described by Semenova *et al*.^26^. Briefly, leader fragments were amplified from genomic DNA with primers specific to *Cas2* gene, RP: CAGGCTCGCGAAATTTCCAAGTGAT, and CRISPR repeat region, FR: CTTGACGGTGTGATGGCC. Then, the fragments were sequenced by a primer-walking method. For XF89b CRISPR region, a clone from a 10 kb fragment library was selected and sequenced by Sanger sequencing. For XM9 CRISPR region, the sequence was obtained from Pacbio Sequel single molecule real-time sequencing and assembly result. For XO21 and XO604 isolates, CRISPR spacer sequence was obtained from a prior study^26^. XO21 and XO604 are Taiwanese isolates reported in prior studies^47,48^. XO604 is also in our sample collection. The last 10 spacers in XO604 CRISPR region were sequenced by Sanger sequencing and showed identical sequence as the prior study^26^. For the other isolates, the sequences were obtained from Quibod *et al*.^17^.

### Optical mapping

Genomic DNA of *Xoo* XF89b and XM42 were isolated and digested with BamHI in optical mapping procedures as described by Kotewicz *et al*.^49^. The detailed procedure followed the manufacturer’s instruction established by Yourgene Bioscience, Taiwan. After the assembly of the restriction map, the comparison of *Xoo* MAFF311018, KACC10331, PXO99A, XF89b, XM42, and *Xoc* BLS256 were analyzed with MapSolver software. Also, the *Xoo* XF89b contigs from the next-generation sequencing result was aligned to optical mapping results with the same software.

### Genome sequencing

*Xoo* XF89b genomic DNA was extracted and randomly sheared. The size of 0.45 kb and 3 kb of sheared DNA were selected on gel and ligated with adaptors. The DNA libraries were sequenced by Illumina GA IIX and MiSeq sequence protocols established and modified by the High Throughput Genomics Core, Academia Sinica. The GA IIX sequencer yielded 33,974,650 reads of the 80 bp mate pair dataset (~1000x), and the MiSeq sequencer yielded 9,509,144 reads of the 250 bp paired end dataset (~480x). The datasets were then assembled by ALLPATHS-LG software. The sheared gDNA was also constructed and sequenced by Roche 454 sequencer. The results yielded 323,730 trimmed reads with an average length of 741.72 bp (~50x). The 454 dataset was assembled by Newbler 2.7.0. The contigs generated from ALLPATHS-LG and Newbler were further aligned and merged by Minimus2 in the AMOS package. The combination of datasets resulted in 224 non-repeated contigs. The order of the contigs was defined with the optical map of *Xoo* XF89b *in silico*. For the highly repeated regions, TAL gene clusters and a CRISPR region, gDNA were partially digested by *SphI*, and 10 kb gDNA fragments were selected on gel. The selected fragments were purified by GenepHlow Gel/PCR Kit (Geneaid, Taiwan). The purified DNA fragments were then repaired by mung bean nuclease. The blunt-end DNA fragments were constructed into Lucigen BigEasy v2.0 Linear Cloning System (pJAZZ-OK blunt vector) according to the manufacturer’s instruction. *E*. *coli* colonies on kanamycin selection plates containing plasmids of TAL genes or a CRISPR region were selected by a PCR screen using locus-specific primers (Supplementary Table S11). The plasmids and gaps of the genome were then filled by PCR and the Sanger sequencing method. The genome sequence is available at NCBI, accession: NZ_CP011532.1. For Xoo XM9, gDNA were sequenced by PacBio Sequel sequencing protocol at Genomics, Taiwan. The raw reads from Sequel sequencing were then assembled by pbsmrtpipe v.0.51.2 and Falcon v.0.3^50^. The assembly generates a single contig. This contig was further corrected by raw reads using Arrow. The annotation of the genome is performed with NCBI prokaryotic genome annotation pipeline v.2.10. The schematic representation of the indel heat map was generated by Circos^51^. In-del regions and a cladogram were generated by Mauve v.2.4.0^52^. The XM9 genome sequence is available at NCBI, accession: CP020334.1. The TALE analysis in this study was mainly based on AnnoTALE classification^33^. RVDs were extracted by the software or derived from a prior study^17^ with manual checking. DisTALE analysis was using QueTAL software^34^.

### RNA-seq and transcriptome analyses

Fourteen-day-old rice cultivar TNG67 was inoculated with Xoo XE3, XF89b, and XM9 using the leaf clipping method. Briefly, *Xoo* isolates were grown in 1/2 TSB medium overnight, and the culture of *Xoo* was diluted to OD_600_ = 1.0 with the same medium. The cultured mediums containing *Xoo* isolates and the 1/2 TSB buffer as a control group were used in the assay. Leaf blades from the clipping site to 5 cm below the clipping site were collected at 7-days post-inoculation. Leaf RNA of the infected rice was extracted by TRIzol RNA isolation reagent (Invitrogen) followed by DNase I digestion. Purified RNAs were further processed by Novogene Illumina Hiseq 4000, PE 150 standard protocol. RNA-seq raw data were further analyzed using CLC workbench software v.10.1.2 (Qiagen). All the configurations for transcriptome analyses by CLC workbench were set to default values. For sequencing data mapping, a rice cDNA library (MSU7) was used as a reference dataset. For the comparison between sample sets, 75^th^ percentile of total mapped reads was used for normalization. The statistics of the transcriptomic data from two biological replicates was performed using the empirical analysis of DGE in CLC workbench. The list of differentially expressed genes was selected by the genes with fold change ≥2 and P < 0.05 in either one of the three control and *Xoo*-inoculated sample sets (XE3 vs. mock, XF89b vs. mock or XM9 vs. mock). The classification of genes in the heatmap was grouped using R v. 3.3.3 with ggplot2 v. 2.2.1^53^. GO enrichment analysis of differentially expressed genes was performed by agriGO v. 2.0^54^ with the Singular Enrichment Analysis (SEA) tool and the *Oryza sativa* MSU7.0 nonTE dataset.

## Electronic supplementary material

Supplementary Information

Supp_Table S1

Supp_Table S2

Supp_Table S3

Supp_Table S4

Supp_Table S5

Supp_Table S6

Supp_Table S7

Supp_Table S8

Supp_Table S9

Supp_Table S10

Supp_Table S11

## Data Availability

All complete genome assemblies and corresponding genome annotations are available at the National Center for Biotechnology Information (NCBI). BioProject: PRJNA284661 (XF89b) and PRJNA378144 (XM9). BioSample: SAMN03729481 (XF89b) and SAMN06480557 (XM9). SRA: XF89b: SRR6510602, SRR6510603, and SRR6510604. XM9: SRR5319797. Accession: XF89b: NZ_CP011532.1, XM9: CP020334.1. Rice RNA-seq data: BioProject: PRJNA378144. BioSample: SAMN08384324. SRA: SRR6513657.
