# Supplementary material for: Analysis of genetic diversity of Xanthomonas oryzae pv. oryzae populations in Taiwan
Source: Sci Rep. 2019 Jan 22;9:316. doi: 10.1038/s41598-018-36575-x (PMC6342995; doi:10.1038/s41598-018-36575-x)
Supplement: Supplementary file 1 — Supplementary Information [file 41598_2018_36575_MOESM1_ESM.pdf]

Supplementary Information

Analysis of genetic diversity of *Xanthomonas oryzae* pv. *oryzae* populations in Taiwan

Chih-Cheng Chien[1,2,3], Mei-Yi Chou[2], Chun-Yi Chen[2,4,5], Ming-Che Shih[1,2,3*]

[1]Molecular and Biological Agricultural Sciences Program, Taiwan International Graduate Program, Academia Sinica, Taipei, 115, Taiwan

[2]Agricultural Biotechnology Research Center, Academia Sinica, Taipei, 115, Taiwan

[3]Graduate Institute of Biotechnology, National Chung-Hsing University, Taichung, 402, Taiwan

[4]Bioinformatics Program, Taiwan International Graduate Program, Academia Sinica, Taipei, 115, Taiwan

[5]Institute of Biomedical Informatics, National Yang-Ming University, Taipei, Taiwan

[*]Correspondence to email address: mcshih@gate.sinica.edu.tw

This file includes:

Figure S1 to S8

Title of Table S1 to S10

Figures S9 to S10 are the original gels/blots in the figures

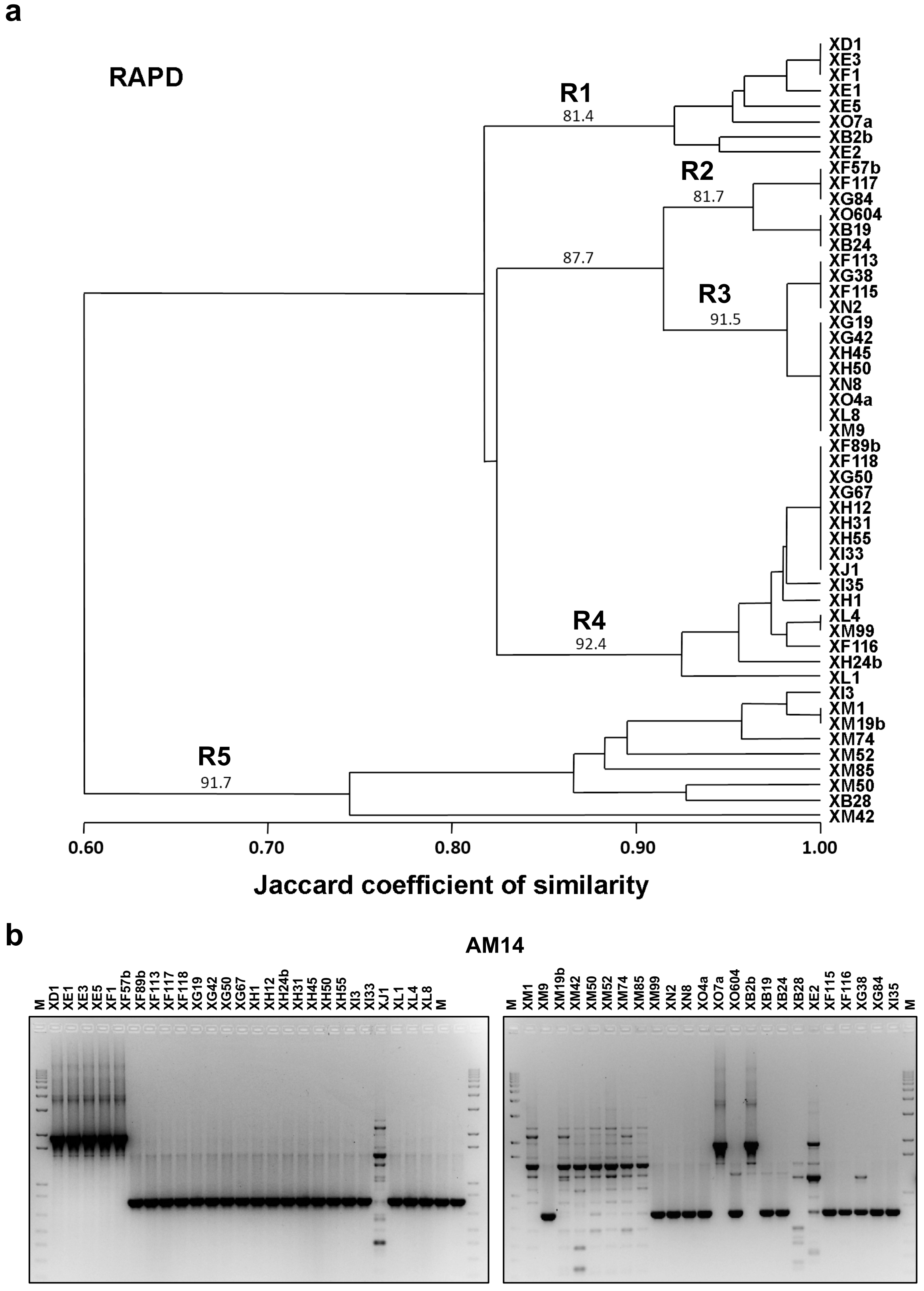

Supplementary Fig. S1. RAPD patterns of Taiwanese *Xoo* isolates
(a) A dendrogram was generated from RAPD binary data by UPGMA clustering. Arabic numbers above the internal branches indicate the significant bootstrap probability (>70%) obtained for 1000 repetitions. R1 to R5 labels represent the major clades in the clustering. (b) PCR-amplified fragments on 1% agarose gel using a RAPD primer AM14. This PCR result represents as an example of the grouping results with 11 RPAD primers.

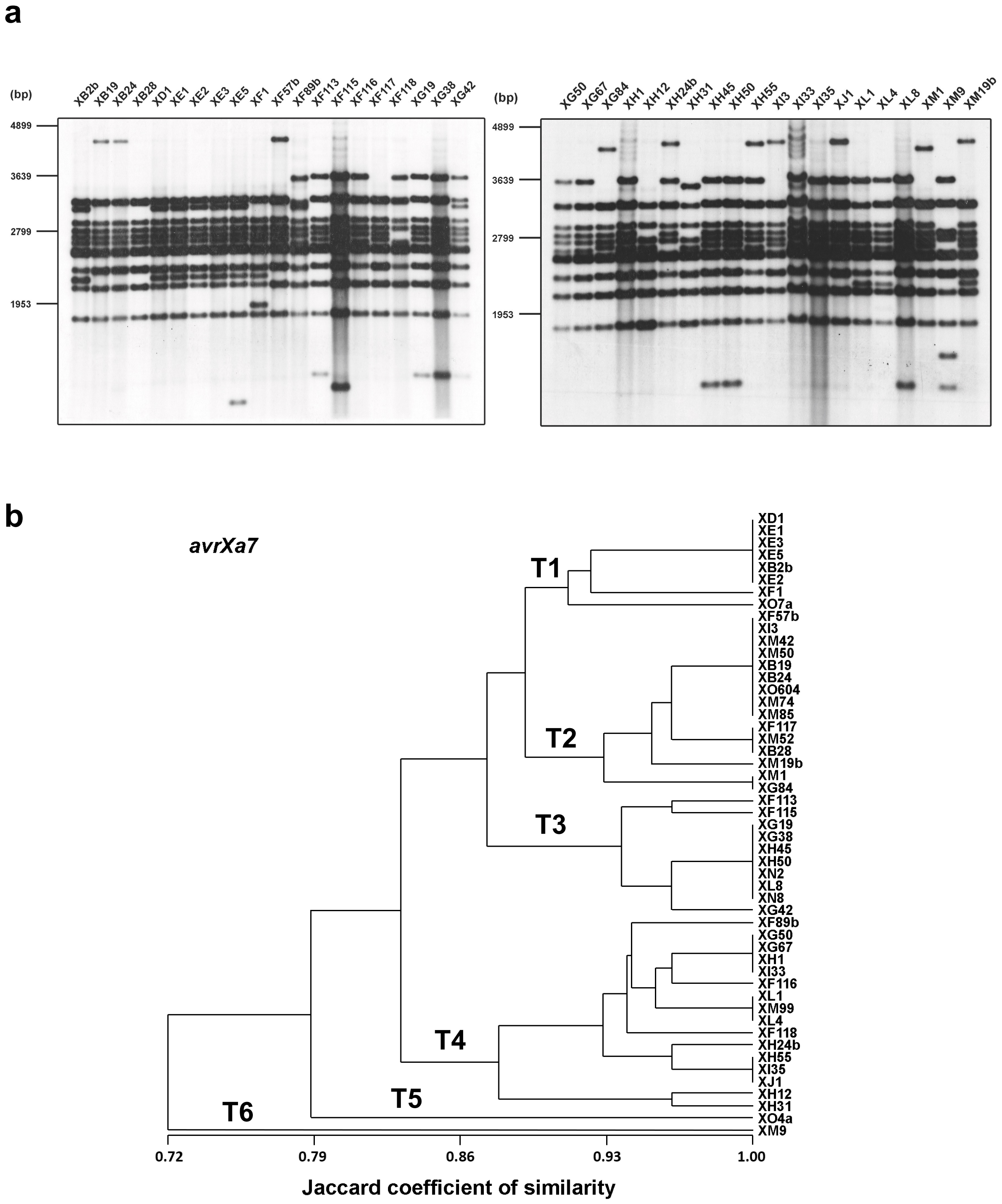

Supplementary Fig. S2. RFLP patterns of Taiwanese *Xoo* isolates
(a) Genomic DNAs of Taiwanese *Xoo* isolates are digested with *SphI* restriction enzyme and analyzed by Southern blotting. The fragments were probed with a 3.1 kb fragment from pZWavrXa7[49]. The restriction patterns indicate TALE gene-containing fragments in the genomes. The results were repeated three or more times with consistent patterns. (b) A dendrogram was generated from RFLP binary data by UPGMA clustering. The different groups of *Xoo* isolates based on the phylogenetic tree are labeled from T1 to T6.

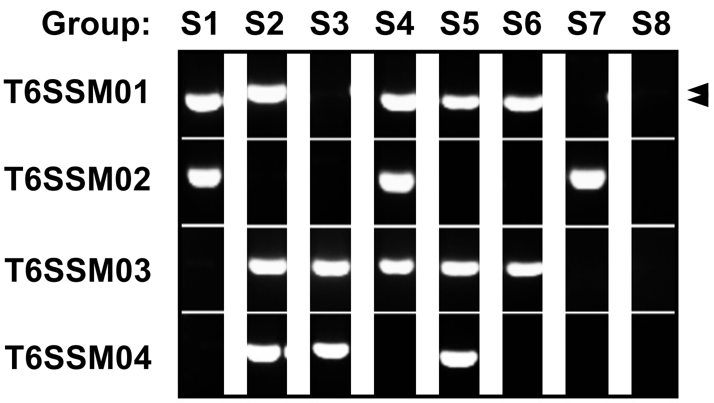

Supplementary Fig. S3. Typical patterns of T6SS-II markers in Taiwanese isolates

S1 to S8 groups showed classifications from Fig. 1c. T6SSM01 marker generates two different sized fragments. The arrowheads indicate the position of these two fragments.

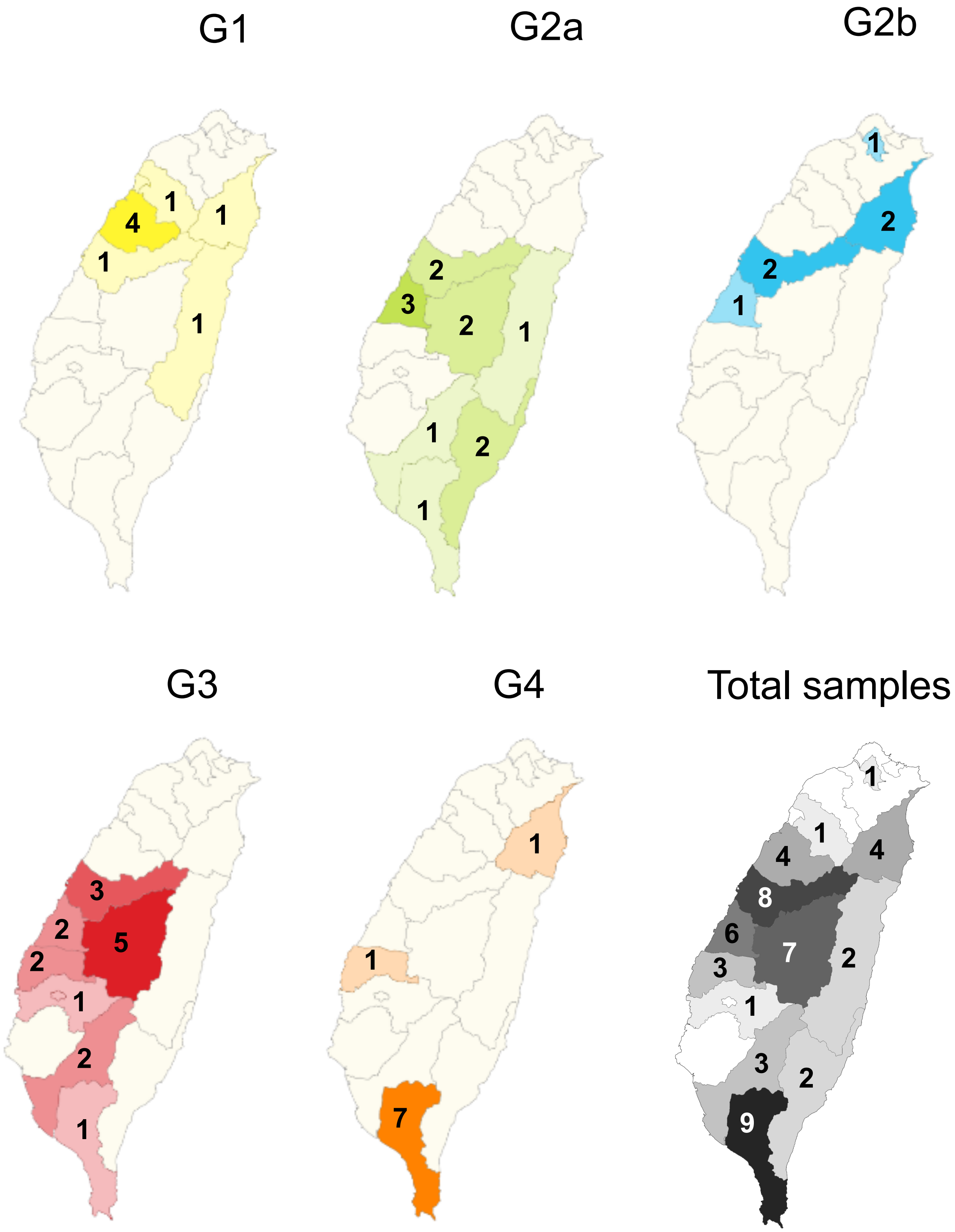

Supplementary Fig. S4. Distribution of *Xoo* clonal populations in Taiwan
Five populations of Taiwanese *Xoo* isolates from 51 collected samples. The Taiwan island was separated into 19 administrative regions, and the *Xoo* isolates collected in each region were classified into 5 identified clonal populations. Numbers in each region represent collected sample numbers, and the darker color represents a greater number of samples in the area.

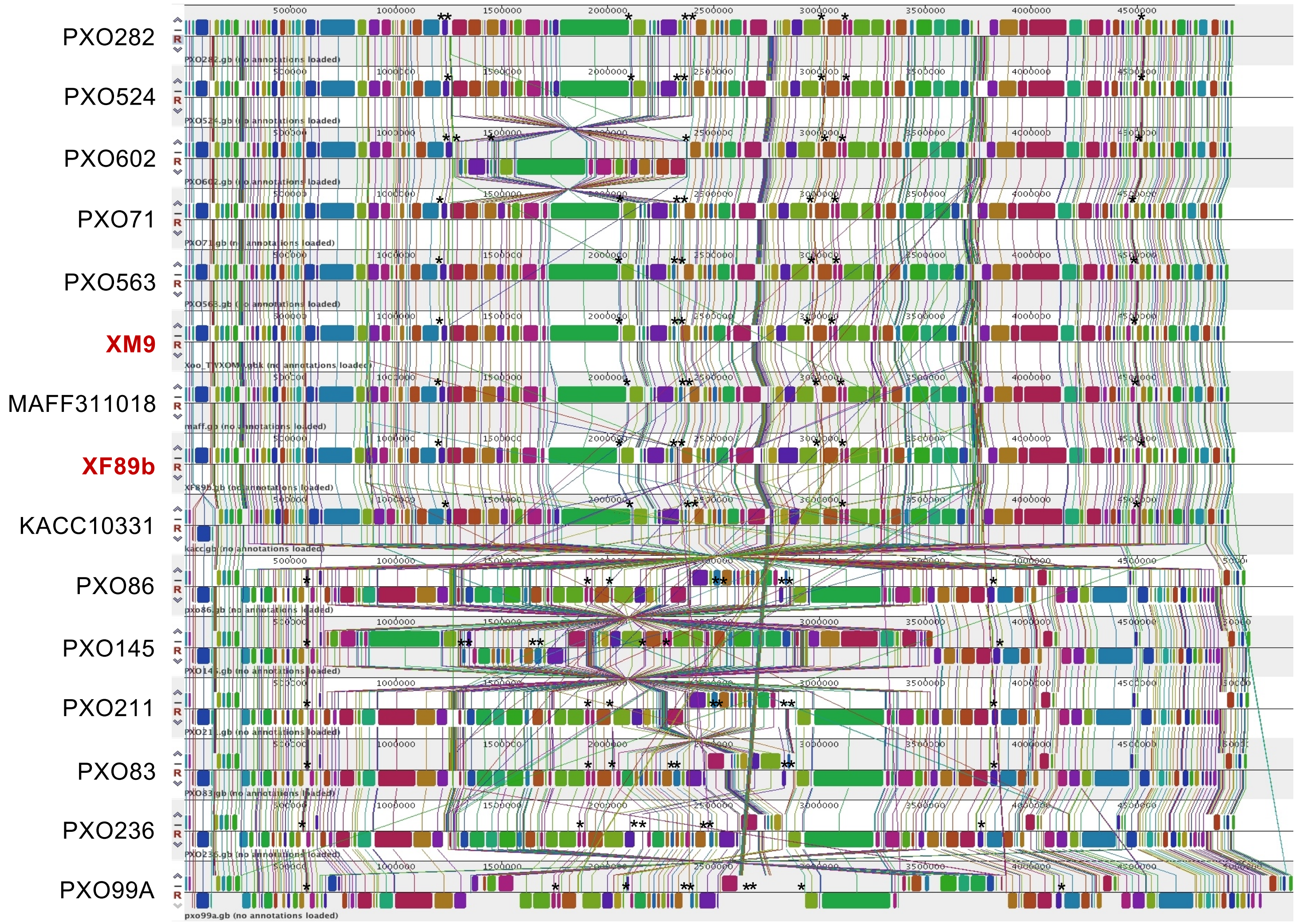

Supplementary Fig. S5. Genome structure of *Xoo* isolates
Genome structure and rearrangement maps generated from MAUVE[52]. Different color blocks represent relatively similar sequences between each isolate. The lines indicate the connection of the similar blocks in each isolate. Two Taiwanese isolates, XM9 and XF89b, were marked in red texts. The stars represent TALE islands in each genome.

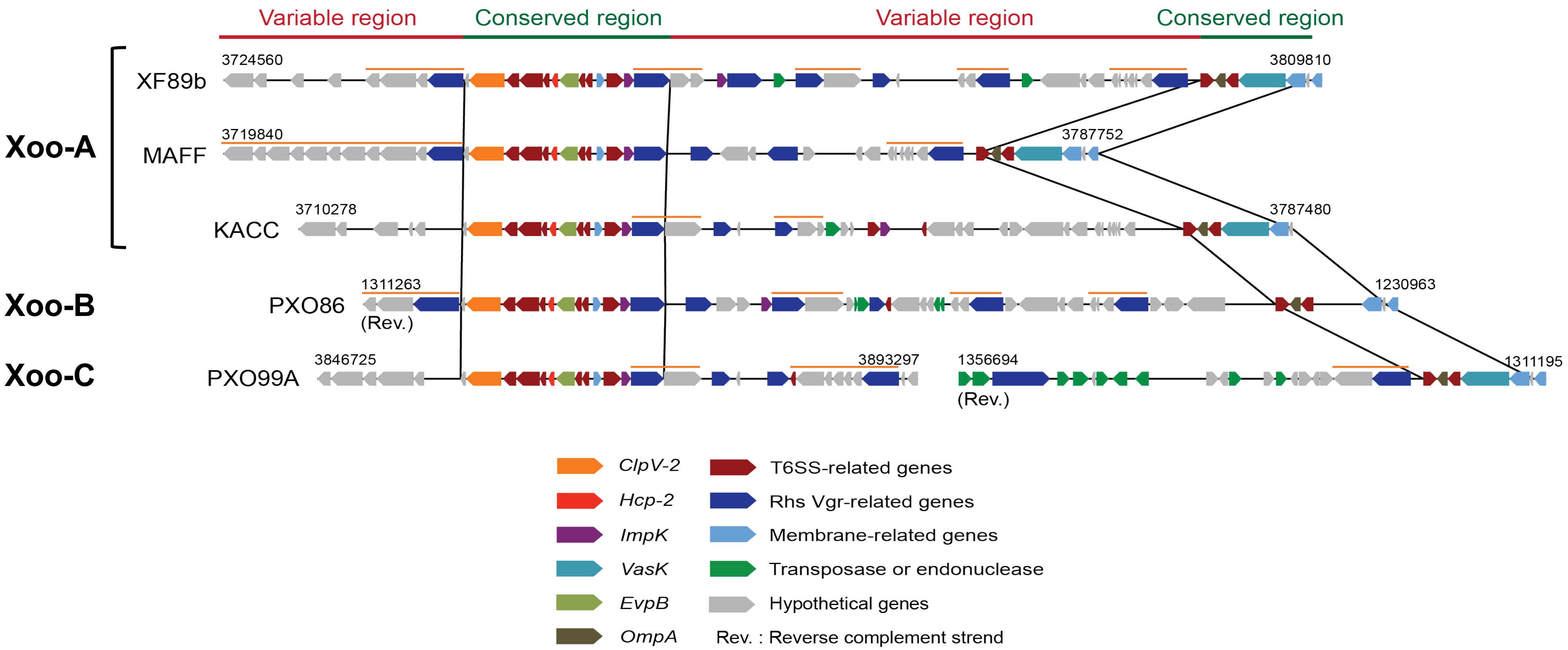

Supplementary Fig. S6. Gene positions of the T6SS-II region in the genomes
A comparison of T6SS-II regions in different *Xoo* isolates. Dark blue arrows represent VgrG-like genes, and the orange line represents putative operons. Genes presented in all isolates, XF89b, MAFF311018 (MAFF), KACC10331 (KACC), PXO86, and PXO99A were marked as conserved regions, and other regions in the T6SS-II region were labeled as variable regions. Numbers at the top of the beginning and the end of each isolate represents positions of the T6SS-II region in the genomes.

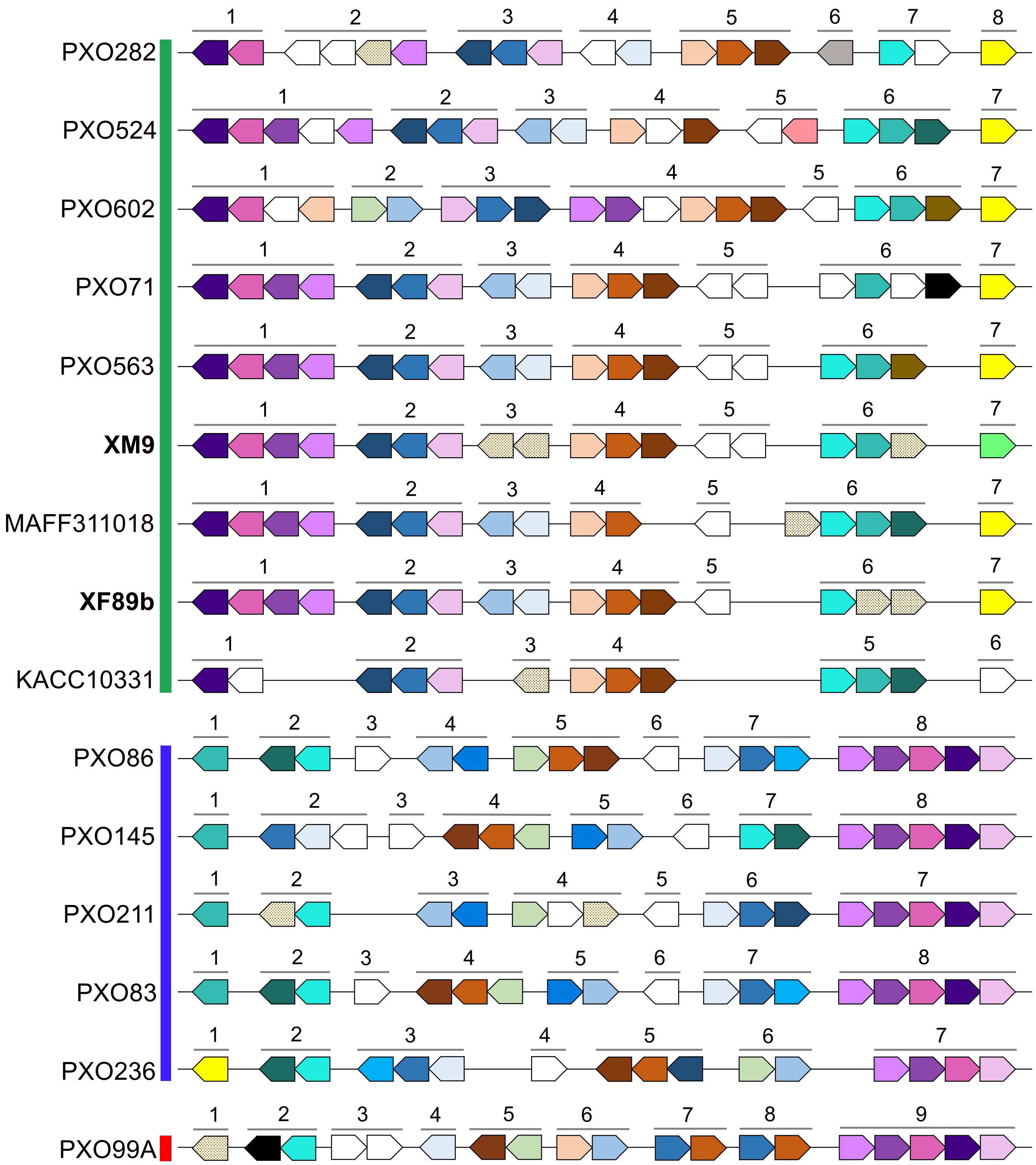

Supplementary Fig. S7. TALE island distribution in each *Xoo* isolate
The arrows indicate the strand direction of TALEs, and different colors represent different TALE classes. White arrows mean pseudo-TALE genes and arrows with dots represent unique TALEs in the comparison. The colored bar on the left indicates classification of *Xoo* isolates based on genome SNPs. Arabic numbers at the top of each TALE gene represent the TALE island numbers in each isolate.

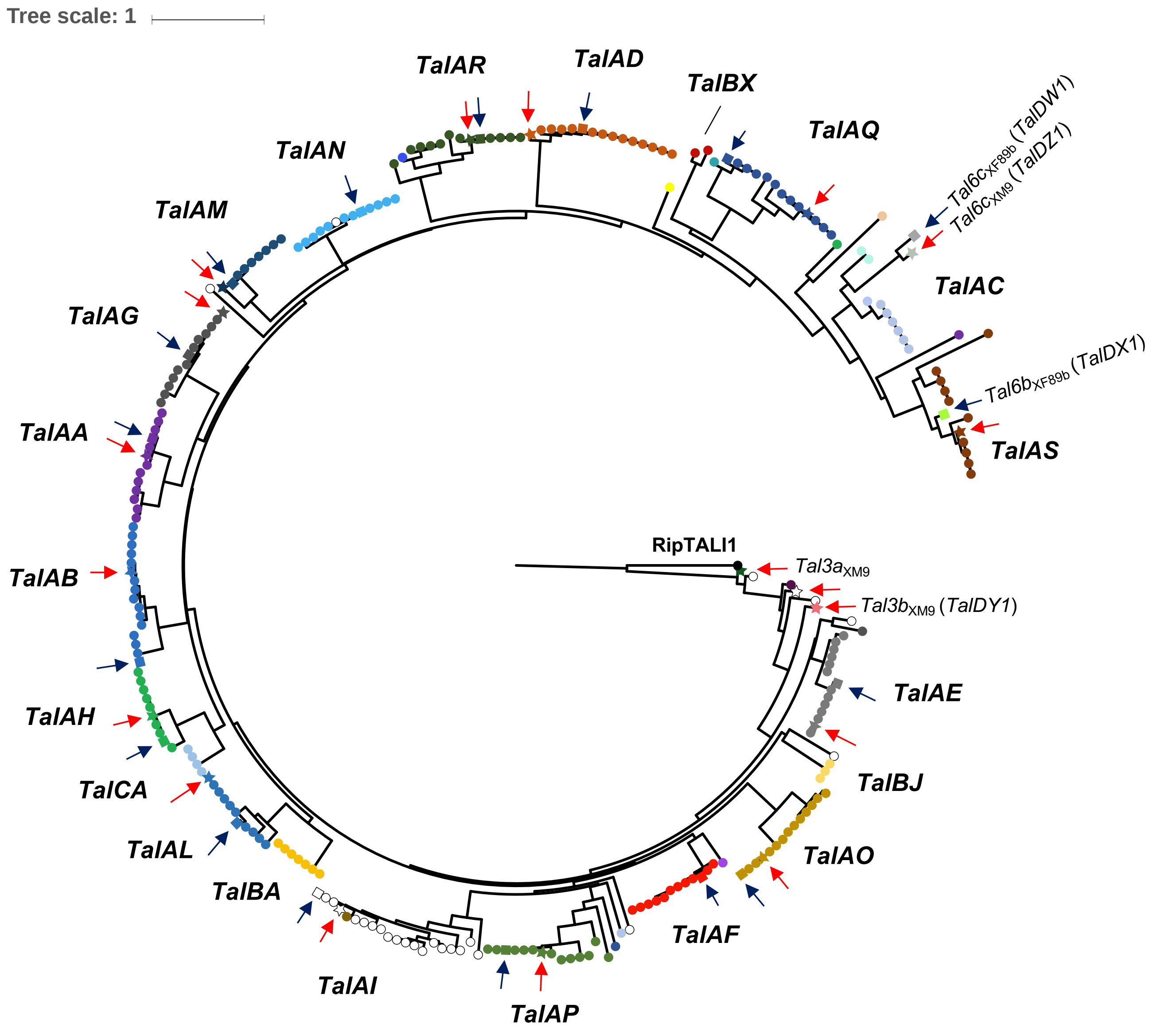

Supplementary Fig. S8. Transcription activator-like effectors class obtained from 15 *Xoo* TALEs

A tree obtained with DisTAL denoting similar classification based on central repeat allelic distribution within each TALE class (see Supplementary Table S4). RipTALI1 is used as an outgroup. The blue arrows and squares represent TALEs in XF89b. The red arrows and stars represent TALEs in XM9.

Supplementary Table S1 Comparison of classification results of 51 Taiwanese *Xoo* isolates

Supplementary Table S2 CRISPR spacer sequence library

Supplementary Table S3 CRISPR spacer order

Supplementary Table S4 TALE classes

Supplementary Table S5 Xop classes

Supplementary Table S6 TALE targets in rice

Supplementary Table S7 Recognition possibility of predicted TALE-targeted gene list in Xoo-A

Supplementary Table S8 Expression values of TALE-targeted TNG67 genes

Supplementary Table S9 Differentially expressed genes in TNG67 after inoculations of XE3, XM9, and XF89b

Supplementary Table S10 Collection information of local *Xoo* isolates

Supplementary Table S11 Primer list used in this study

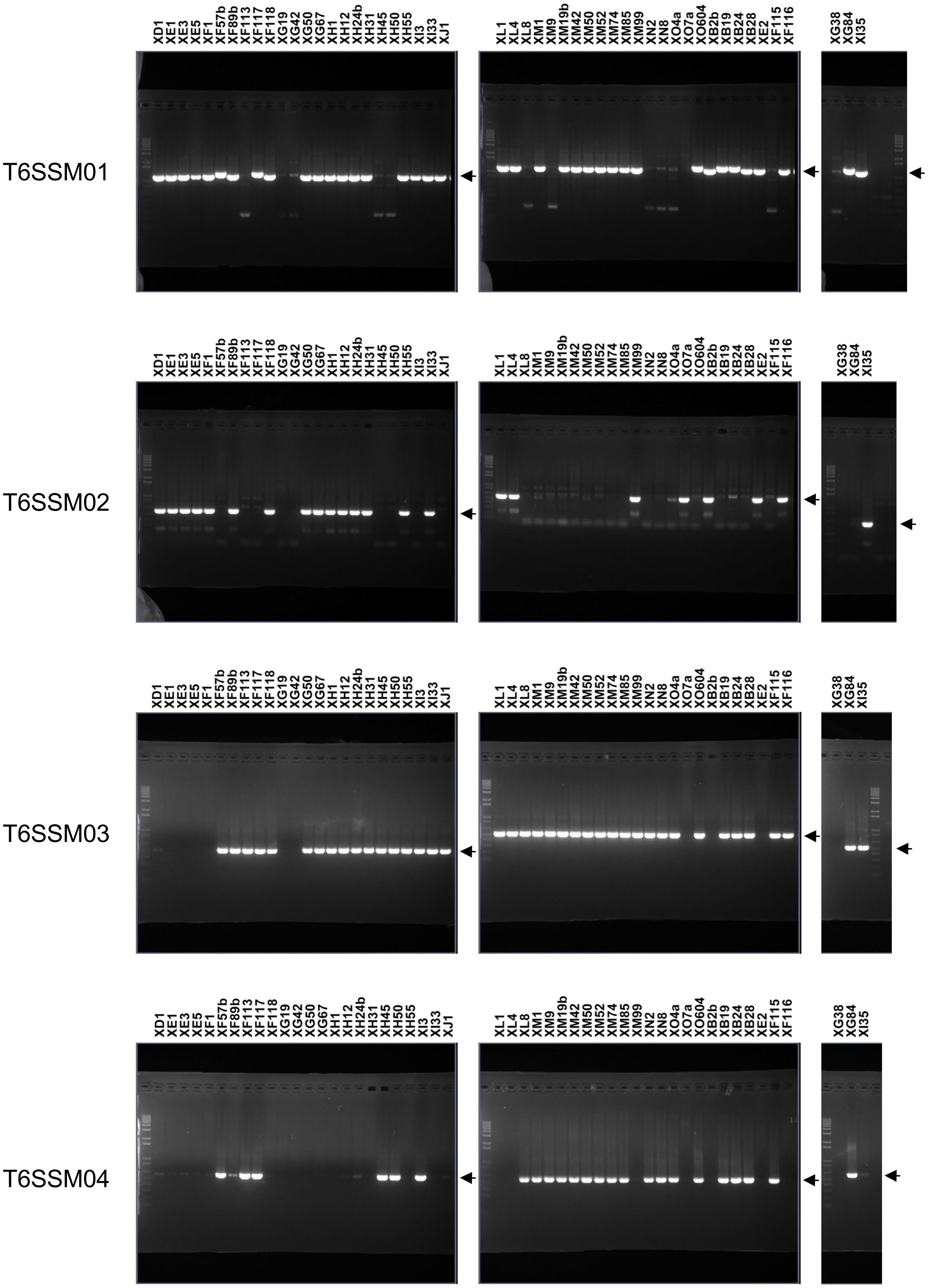

Supplementary Fig. S9. Original gels in Fig. 1c
The arrows represent the bands shown in Fig.1c

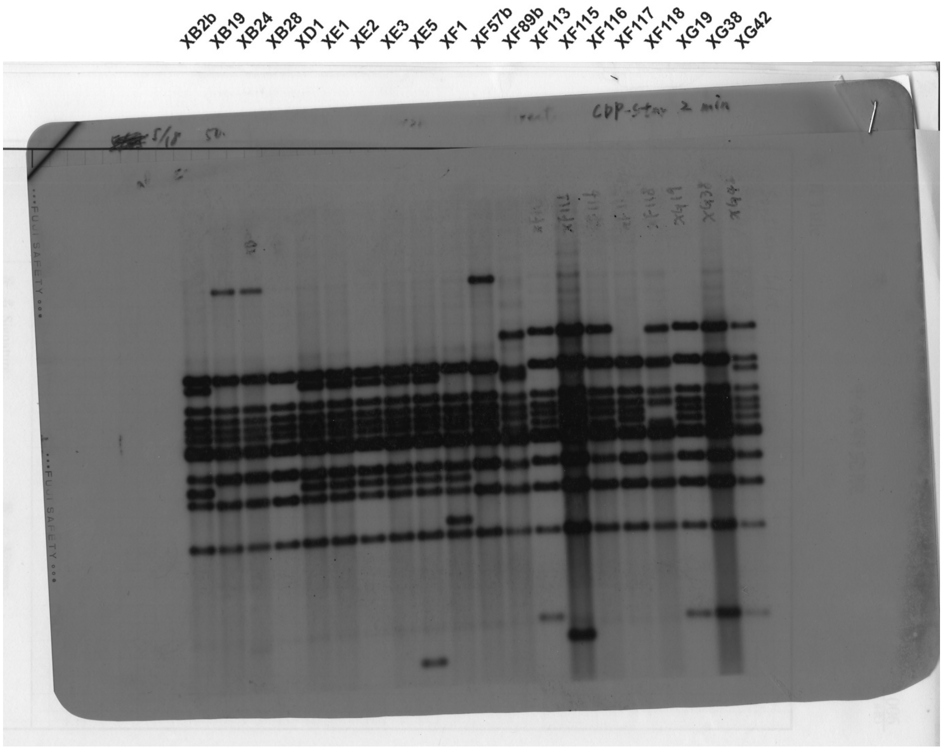

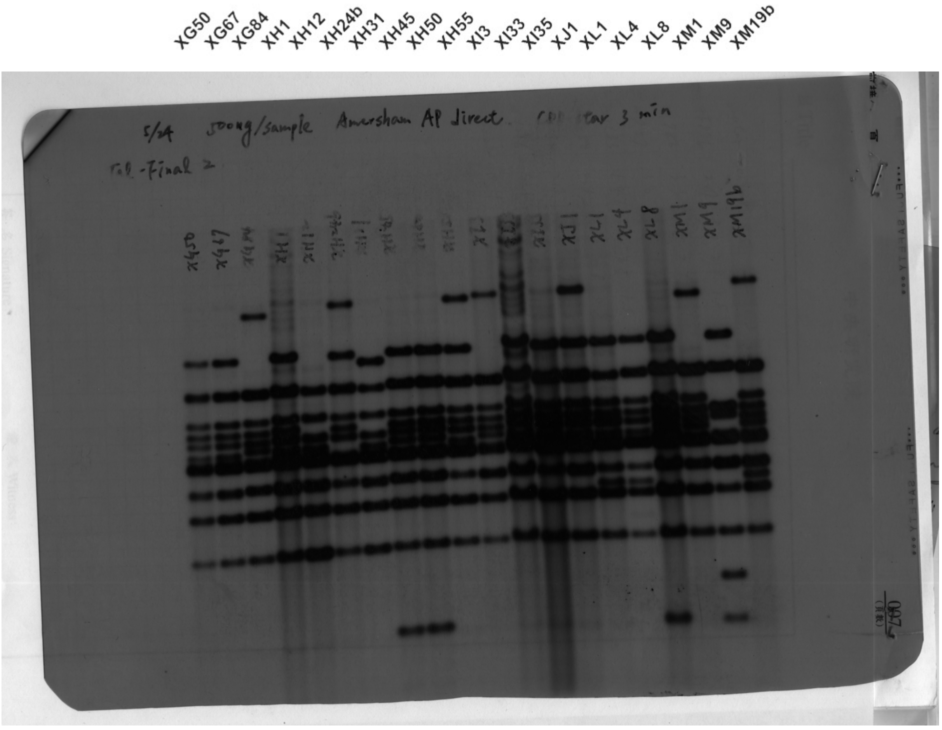

Supplementary Fig. S10. Original blot films in Fig. S2
